# Transcription factor enrichment analysis (TFEA) quantifies the activity of multiple transcription factors from a single experiment

**DOI:** 10.1038/s42003-021-02153-7

**Published:** 2021-06-02

**Authors:** Jonathan D. Rubin, Jacob T. Stanley, Rutendo F. Sigauke, Cecilia B. Levandowski, Zachary L. Maas, Jessica Westfall, Dylan J. Taatjes, Robin D. Dowell

**Affiliations:** 1 Department of Biochemistry, University of Colorado, Boulder, CO USA; 2 BioFrontiers Institute, University of Colorado, Boulder, CO USA; 3 Computational Bioscience Program, Anschutz Medical Campus, University of Colorado, Aurora, CO USA; 4 Department of Molecular, Cellular and Developmental Biology, University of Colorado, Boulder, CO USA; 5 Department of Computer Science, University of Colorado, Boulder, CO USA

**Keywords:** Transcriptional regulatory elements, Statistical methods

## Abstract

Detecting changes in the activity of a transcription factor (TF) in response to a perturbation provides insights into the underlying cellular process. Transcription Factor Enrichment Analysis (TFEA) is a robust and reliable computational method that detects positional motif enrichment associated with changes in transcription observed in response to a perturbation. TFEA detects positional motif enrichment within a list of ranked regions of interest (ROIs), typically sites of RNA polymerase initiation inferred from regulatory data such as nascent transcription. Therefore, we also introduce *muMerge*, a statistically principled method of generating a consensus list of ROIs from multiple replicates and conditions. TFEA is broadly applicable to data that informs on transcriptional regulation including nascent transcription (eg. PRO-Seq), CAGE, histone ChIP-Seq, and accessibility data (e.g., ATAC-Seq). TFEA not only identifies the key regulators responding to a perturbation, but also temporally unravels regulatory networks with time series data. Consequently, TFEA serves as a hypothesis-generating tool that provides an easy, rigorous, and cost-effective means to broadly assess TF activity yielding new biological insights.

## Abstract (web summary)

Rubin et al. introduce transcription factor enrichment analysis (TFEA), a new motif enrichment method specifically aimed at maximizing the informative nature of differential RNA polymerase initiation data. It provides an easy, rigorous, and cost-effective analysis aimed at deciphering the temporal and mechanistic details of complex regulatory networks.

## Introduction

The cellular response to everything from environmental stimuli to development is orchestrated by transcription factors (TFs). Therefore, when transcription changes, one important objective is to infer which TFs are causally responsible for the observed changes. TFs bind to DNA at preferred sequence-specific recognition motifs and ultimately alter transcription nearby. Extensive DNA-protein binding has been measured by chromatin immunoprecipitation (ChIP)^1–3^, leading to large collections of high-quality sequence recognition motifs^3–5^. Unfortunately, acquisition of protein–DNA binding is not sufficient for understanding regulation, as many binding sites do not lead to altered transcription nearby^6–8^.

In an effort to causally link a TF to observed transcription changes, binding data is often combined with expression, typically measured by RNA-seq^9–11^. However, the success of this approach is limited. Fundamentally, the difficulty lies not in the binding data, but rather in the use of steady-state RNA-seq to assay expression. RNA-seq levels reflect both transcription and degradation^12–14^, e.g., both newly created and long-lived RNAs contribute to the measurement^15,16^. Hence, RNA-seq is, at best, only an indirect measure on transcription. In addition, RNA-seq data is dominated by the most abundant RNAs, rather than those that are most recently made. Thus, after ribosomal RNAs, the dominant signal is protein-coding genes, which are highly stable processed transcripts. In addition, to infer TF activity, one must solve the assignment problem^9^—namely linking TF binding sites to stable gene transcripts, which are often both positionally (in the genome) and temporally (RNA processing) distant^17^.

Nascent transcription^18,19^ circumvents the assignment problem. Nascent transcription assays measure bona fide transcription, prior to RNA processing. Thus, changes in transcription induced by TFs can be detected within minutes^20^. Conveniently, a TF’s regulatory activity has been shown to alter RNA polymerase initiation immediately proximal to sites of TF binding^21–23^. The majority of altered RNA polymerase initiation sites are at transcription regulatory regions (e.g., enhancers) -not at genes^24^. Thus, by using all polymerase initiation sites (both at enhancers and genes) rather than just the target gene, the assignment problem is sidestepped^25^. Enhancer RNAs (eRNAs) are highly transient unstable transcripts that are essentially undetectable in RNA-seq, yet effectively serve as markers of TF activity. Consequently, our previous work demonstrated the ability to directly infer causal TF activity from changes in RNA polymerase initiation observed in nascent transcription assays^26^.

Despite recent improvements to the protocols^27–29^, nascent transcription assays are less popular than other genomic assays, likely due to their perceived difficulty. Luckily, a variety of popular high throughput assays also have a relationship with RNA polymerase initiation and therefore could serve as proxies to nascent transcription. For example, cap-associated approaches, such as CAGE, target the 5${}^{\prime}$ cap of transcripts^30–32^ and are therefore a viable alternative to nascent transcription. However, CAGE provides only a subset of RNA polymerase initiation sites, biased to stable transcripts^31^. In contrast, transcription arises from only a subset of nucleosome-free regions, therefore chromatin accessibility data indirectly informs on the locations of transcription initiation. Likewise, some histone marks have been associated with actively transcribed regions, such as H3K27ac and H3K4me1/2/3^33^. In principle, all of these methods provide some information on sites of RNA polymerase initiation, but with distinct detection limits, positional precision, and temporal fidelity. To leverage these datasets, a motif enrichment method is needed that seamlessly handles the uncertainty inherent in using an approximation to RNA polymerase initiation.

Therefore, we introduce TF enrichment analysis (TFEA), a motif enrichment method specifically aimed at maximizing the informative nature of differential RNA polymerase initiation data, where positional information is critically important^26,34^. TFEA not only accounts for the position of the motif relative to transcription initiation, but also accounts for the magnitude of transcription change (i.e., differential signal)^35–37^. Critically, TFEA is robust to noise in both of these sources of information (position and signal) and therefore can be applied to a number of different regulatory datasets. Finally, TFEA is fast, computationally inexpensive, and designed with the user in mind, as we provide an easy-to-use command-line interface, container images (Docker and Singularity), and an importable Python 3 package. TFEA provides easy downstream analysis aimed at deciphering the temporal and mechanistic details of complex regulatory networks.

## Results

### Overview

**Fig. 1:**
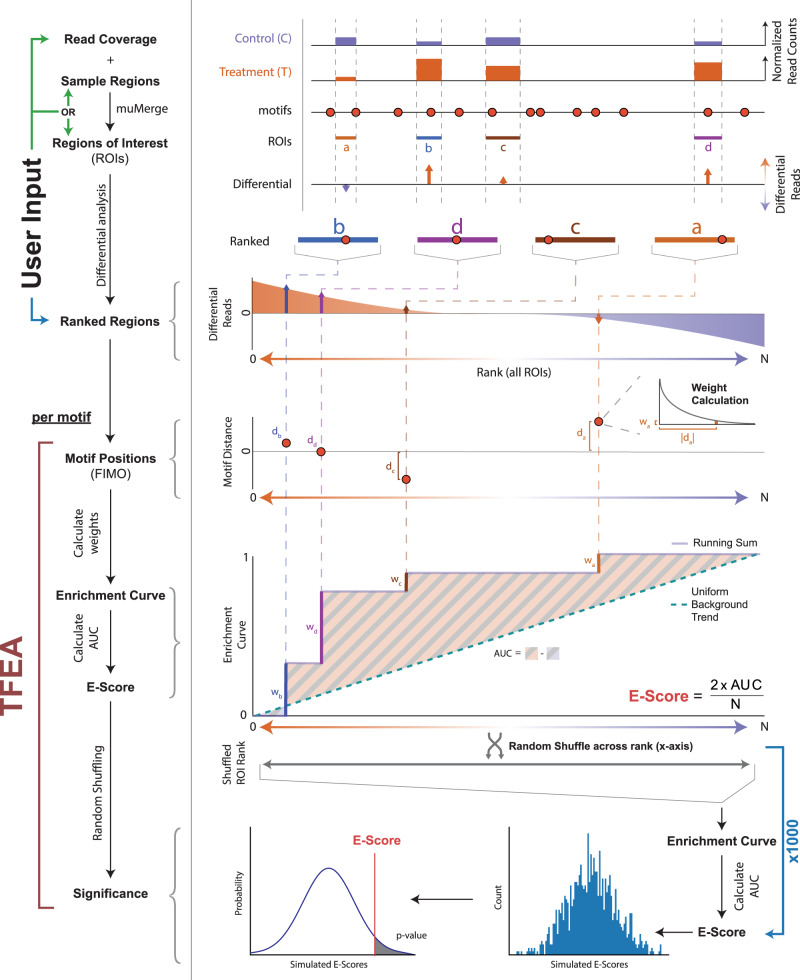
TFEA calculates motif enrichment using differential and positional information. The TFEA pipeline requires, minimally, a ranked list of ROIs (control in blue, treatment in orange). Optionally, a user may provide raw read coverage and regions (ROI, colored boxes labeled **a**–**d**), in which case TFEA will perform ranking using DESeq^2,43^ analysis. With a set of ranked ROIs (orange up, blue down), TFEA analyzes motif enrichment for each motif provided (red circles). For each motif, positions are determined by FIMO scans, and an enrichment curve is calculated by weighting each motif instance (with weight *w*_*i*_, using an exponential decay as a function of the motif distance *d*_*i*_ from the region center) and adding this value to a running sum. An *E*-score is calculated as 2 * AUC, e.g., the area under the enrichment curve between the running sum and a uniform background (dashed line), and scaled by the number of motif instances *N*. For statistical significance, the ROI rank is randomly shuffled 1000 times, and *E*-scores are recalculated for each shuffle. The true *E*-Score is then compared to the distribution of *E*-Scores obtained from the shuffling events. For example, the output of TFEA, see Supplementary Fig. 1 and Supplementary Fig. 2.

TFEA seeks to identify which TF(s) are causally responsible for observed changes in transcription between two data sets. An overview of this procedure is shown in Figure 1 (see Supplementary Figs. 1 and 2 for example outputs). Briefly, TFEA takes as input a set of RNA polymerase initiation regions and ranks them, preferably by changes in transcription levels between the two conditions. The ranked list is then used to calculate a TF motif enrichment score, which incorporates not only the differential transcription signal at initiation sites but also the distance to the nearest motif instance. The TF enrichment score is then compared to the distribution of expected scores, empirically derived, to assess the statistical significance of the TF motif enrichment.

Importantly, for each cell type and condition, RNA polymerase initiates transcription from a distinct set of locations. Biologically, each RNA polymerase initiation event corresponds to an individual transcription start site (TSS). However, most sites of initiation occur in regions of bidirectional transcription with two closely, oppositely oriented TSSs^24,38,39^. Many assays are unable to distinguish between the two TSSs within an RNA polymerase loading zone^25^ (also see Methods section “Regions of interest”). Therefore, without loss of generality, we assume each assay provides a set of regions of interest (ROI) where each region corresponds to either a single TSS or the midpoint between bidirectional TSSs. Each ROI provides a point estimate (the midpoint of the region) and uncertainty on that reference point (width of the region). Because initiation sites are inferred directly from data, they must first be combined across replicates and conditions in a manner that maintains high fidelity on the position of RNA polymerase initiation. Thus we first introduce and evaluate *muMerge*, a method of combining ROI.

### *muMerge*: Combining genomic features from multiple samples into consensus regions of interest

**Fig. 2:**
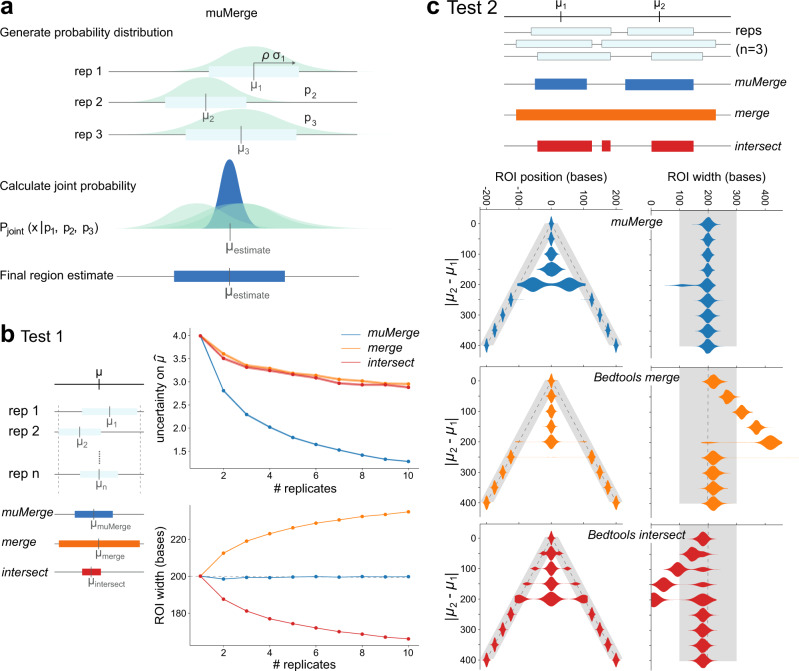
*muMerge* precisely combines multiple samples into consensus ROIs. **a** A schematic for the *muMerge* method. Each sample region (light blue box) is represented by a probability distribution (green, Eq. (1), with centers *μ*_*i*_ and stdev *ρσ*_*i*_), which are combined into a joint probability distribution (dark blue peak, Eq. (2)) from which the final ROI estimates are inferred (dark blue bar). **b** Test 1 demonstrates the position and width accuracy of a calculated ROI for a single locus, *μ*, as the number of sample replicates are increased (from one to ten). The three methods, *bedtools merge* (orange), *bedtools intersect* (red), and *muMerge* (dark blue), for generating ROIs from multiple samples are compared. With *muMerge* the uncertainty on $\hat{\mu }$ (i.e., the standard deviation of the distance between the ground truth position, *μ*, and its estimate, $\hat{\mu }\in \{{\mu }_{muMerge},{\mu }_{merge},{\mu }_{intersect}\}$) decreases quickly while the estimated ROI width remains essentially constant. The standard error, indicated by colored shading, is less than the line width in most cases. **c** Test 2 demonstrates the precision of the calculated ROI for two closely spaced loci, *μ*_1_ and *μ*_2_, as the spacing between them is increased. In this case, *muMerge* transitions from a single locus to two distinct loci more gradually (violin plots, ROI position) and the estimated ROI widths do not deviate from the expected value (violin plots, ROI width), unlike *merge* and *intersect*. In all cases, expected value and variance used for the simulations is indicated by dashed grey lines and shading, respectively. For further detail on the results of Test 1 and 2 and how the simulations were performed, see Supplementary Fig. 4 and Methods *section "muMerge*: Simulating replicates for calculation of ROIs".

A key challenge in defining a set of consensus ROIs is retaining positional precision when combining region estimates that originate from different samples (replicates and/or conditions). To this end, we developed a statistically principled method of performing this combination called *muMerge*. In short, *muMerge* treats the ROIs from each sample as probability distributions and combines these across samples, according to whether they are replicates or different conditions, to produce a joint probability distribution that describes the highest likelihood position for polymerase initiation (see Fig. 2a, Supplementary Fig. 3 and “Methods” section “Defining ROIs with *muMerge*” for full details).

In order to demonstrate the efficacy of *muMerge*, we compare its performance to two common methods for combining regions across multiple samples—merging all samples (e.g., with *bedtools merge*) and intersecting all samples (e.g., with *bedtools interesect*). We performed two tests using simulated data (Fig. 2b, c; Supplementary Fig. 4). For each replicate, we performed 10,000 simulations of sample regions for a single locus and calculated the average performance.

Using the simulated regions, we first evaluate each methods’ precision as the number of replicates increases. In Fig. 2b, we observe that as the number of replicates increases *muMerge* converges on the correct theoretical locus position (*μ*) more quickly than the other two methods (i.e., the vertical axis “uncertainty on $\hat{\mu }$” is the standard deviation of the distance between *μ* and its estimate ($\hat{\mu }$), which is computed from all 10,000 simulations), while still maintaining the correct width for the region.

The second test sought to evaluate the accuracy of these methods when inferring two closely spaced loci, with increasing distance between those loci (Fig. 2c). While closely spaced loci are challenging to distinguish, we observe that *muMerge* smoothly transitions from calling a single inferred locus (when *μ*_1_ and *μ*_2_ are too close to be resolved) to two distinct loci. In contrast, the *merge* and *intersect* methods show abrupt transitions that follow increasingly poor ROI width estimates (Fig. 2c). These tests quantitatively demonstrate the benefit of *muMerge* over the other two methods using simulated data. A comparison using experimental ChIP-seq data^2,40^, where the position of the TF motif instance is used as ground truth, further supports this conclusion (Supplementary Figs. 5 and 6). Examples of the output from all three methods on ChIP-seq data are shown in Supplementary Fig. 7.

### TF enrichment analysis

Armed with the defined set of ROIs, the goal of TFEA is to determine if a given TF motif shows positional enrichment preferentially at regions with the higher differential signals. Therefore an enrichment metric is necessary that accounts for not only the positional enrichment of the motif but also the underlying changes in transcription (Fig. 1). The enrichment metric builds on previous work^26^ but provides substantial improvements by eliminating arbitrary cutoffs and refines the sensitivity to motif position, which is not present in other methods^41^.

In prior work, we assessed the enrichment of motifs relative to positions of RNA polymerase initiation using a co-occurrence metric hereafter referred to as a motif displacement score (MD-Score)^26^. The MD-Score is simply the ratio of TF sequence motif instances within 150 bp radius of ROI midpoints, relative to a larger local 1500 bp radius (see Supplementary Fig. 8 for full details). Unfortunately, the MD-Score approach not only ignored alterations in transcript levels (see Supplementary Fig. 9) but also utilized arbitrary distance thresholds to classify motif proximity in a binary fashion. To account for changes in transcription levels, we subsequently ranked ROIs by differential signal (e.g., transcription) before performing motif displacement calculations within these regions^37^. This method, referred to as differential motif displacement analysis (MDD) compared MD-Scores between the set of differentially transcribed regions to the MD-Score obtained from regions whose transcription is unchanged (see Supplementary Fig. 10 for full details)^36,37^. Unfortunately, the MDD-Score approach introduces an additional arbitrary threshold (e.g., to classify regions as differentially transcribed or not) and still uses the arbitrary motif distance thresholds set by the original MD-Score approach. For TFEA we sought a method that eliminates the reliance on arbitrary cutoffs.

With TFEA, we begin by leveraging the statistically robust, gold standard DESeq package^42,43^ to rank regions based not only on the differential *p* value but also the direction of fold change. Each region of interest then contributes positively to the enrichment curve in a weighted fashion. These weights are determined by the distance of the motif to the reference point using an exponential function to favor closer motifs. The subsequent enrichment score (*E*-score in Fig. 1) is proportional to the integrated difference between the observed and background enrichment curves, calculated as the area under the curve (AUC) in Fig. 1 (see Eq. (8)). The background (null) enrichment curve assumes uniform enrichment across all ROIs, regardless of the differential signal.

By default, TFEA accounts for the known GC bias of enhancers and promoters by incorporating a correction to the enrichment score (Supplementary Fig. 11). Once *E*-scores for all TFs have been calculated, we fit a linear regression to the distribution of these scores as a function of motif GC-content. Corrected *E*-scores are then calculated from the observed *E*-score with the y-offset observed from the linear regression fit (see Eq. (10)). This GC bias correction can be optionally turned off.

Subsequently, we assess the significance of the enrichment score by comparison to randomized ROI order, similar to GSEA^44^. To this end, we generate a null distribution of enrichment scores from random permutations, shuffling the rank order of regions and recalculating the *E*-score for each shuffled permutation. The final significance of the enrichment score is then calculated from the *Z*-score, using the Bonferroni correction to account for multiple hypothesis testing. In this manner, TFEA provides a statistically robust and principled way of calculating motif enrichment that accounts for both differential transcription and motif position without arbitrary distance or differential transcription cutoffs.

### Differential transcription signal improves motif inference over positional information alone

**Fig. 3:**
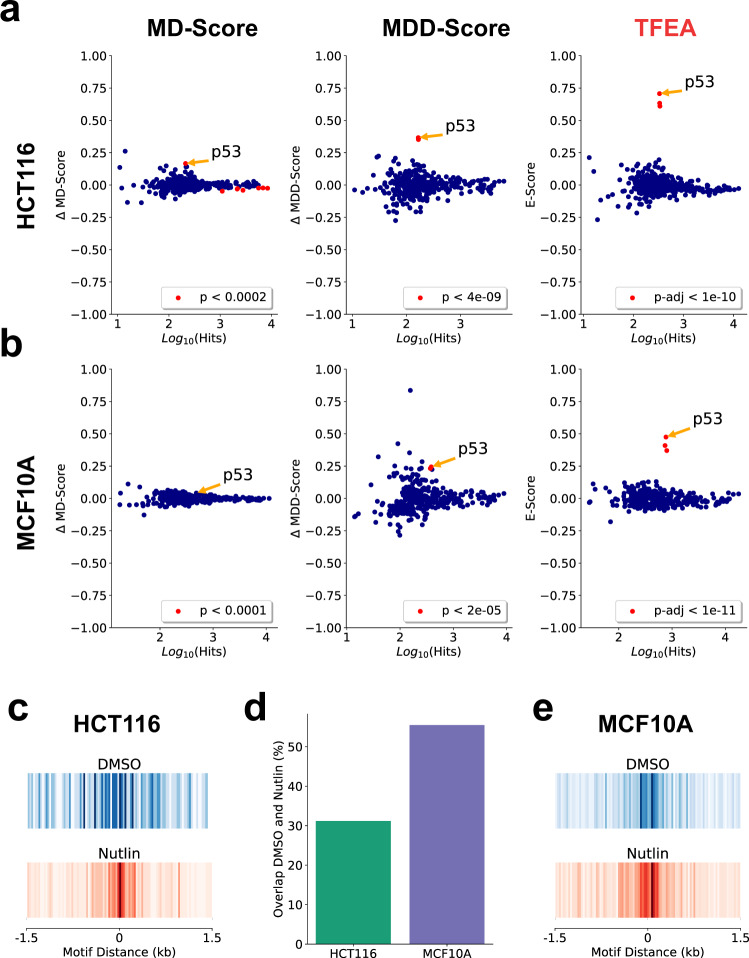
TFEA improves the detection of p53 following Nutlin-3a treatment. **a** Application of the MD-Score, MDD-Score, and TFEA to GROSeq data in HCT116 cells with 1 h Nutlin-3a or DMSO treatment^1^. MA plots contrast a number of regions with motif (*x*-axis) to the change in each score (*y*-axis). Each dot is a distinct position-specific scoring matrix (e.g., TF) with significant changes highlighted in red. Cutoffs determined by comparing untreated replicates (see Supplemental Fig. 12). **b** Application of the MD-Score, MDD-Score, and TFEA to PRO-Seq data in MCF10A cells with 1 h Nutlin-3a or DMSO treatment. **c** Motif displacement distribution plot of TP53 motif instances within 1.5 kb of all ROI in either DMSO (blue) or Nutlin-3a (red) (as a heatmap, darker indicates more motif instances). **d** Percentage overlap of TP53 motif instances within 150 bp of DMSO and Nutlin-3a ROIs. **e** Similar to (**c**) but in MCF10A cells. See Supplementary Data 1 for a complete list of accession numbers for data utilized.

To assess the effectiveness of the TFEA method, we first compared its performance to both the MD-Score^26^ and MDD-Score^36,37^ approaches. We examined a dataset in which a 1-h Nutlin-3a treatment of HCT116 cells is used to activate *TP53*^22^. For all methods, sites of RNA polymerase loading and initiation were determined from GRO-seq data^22^ using the Tfit algorithm, which leverages a mathematical model of RNA polymerase II behavior to identify RNA polymerase loading zones directly from patterns in the data^45^. These sites were then combined using *muMerge* to identify ROIs. For all methods, the significance threshold utilized was determined by comparing within treatment replicates (e.g., DMSO to DMSO) and identifying the score at which no changes are detected (see Supplementary Fig. 12). Using these per method thresholds, we recover *TP53* from all three approaches (Fig. 3a). Notably, by including differential transcription information, the signal-to-noise ratio of *TP53* detection is drastically improved—modestly in the case of MDD and dramatically for TFEA.

We next sought to determine whether TFEA could infer the responsible TF when the underlying changes in transcription were predominantly alterations in existing transcript levels. For this test, we relied on the fact that *TP53* response in epithelial cells depends on the *TP53* family member *TP63*^46^. Because *TP53* and *TP63* have nearly identical motifs, we reasoned that the presence of a constitutively active *TP63* would result in elevated basal transcription proximal to *TP53*/*TP63* motifs. To test this hypothesis, we performed PRO-seq on MCF10A cells after 1-h treatment of either DMSO (control) or Nutlin-3a and applied all three methods to the resulting data.

Consistent with the constitutive activity of *TP63*, we observed no change in the *TP53* motif by MD-Score analysis (Fig. 3b, left). This is due to a larger fraction of ROIs having pre-existing transcription, prior to Nutlin-3a exposure, in MCF10A relative to HCT116 cells (Fig. 3c–e, Supplementary Fig. 13). While the MDD-Score method recovers *TP53* (Fig. 3b, middle), TFEA drastically improves the signal of the *TP53* motif relative to the distribution of all other motifs (Fig. 3b, right). For a more detailed analysis of *TP53* after Nutlin-3a in HCT116 and MCF10A, see Supplementary Figs. 14 and 15.

### TFEA improves motif enrichment detection by incorporating positional information

We next sought to quantify the performance of TFEA with varying degrees of signal, background, and positional information. As a reference point, we leveraged the widely used MEME-Suite component AME, which quantifies motif enrichment by fitting a linear regression to ranked ROIs as a function of motif instances (Supplementary Fig. 16)^47^. Importantly, AME does not utilize positional information.

To compare the two methods, we required biologically representative data sets with known motif enrichment, so that error rates could be readily calculated. To this end, we utilized the sites of RNA polymerase initiation detected in untreated GRO-seq datasets of HCT116 cells^22^ as the base set of ROIs. As there is no second dataset for this comparison, the ROI was then arbitrarily ranked to mimic a pattern of differential transcription. Subsequently, specific instances of the HOCOMOCO^48^ obtained *TP53* motif was embedded via sequence replacement into the ordered ROI list. Importantly, the position and frequency of embedded motifs (e.g., true signal) are varied to simulate distinct TF motif enrichment patterns (see Supplementary Fig. 17 and Methods section “TFEA: Simulated motif enrichment”), allowing us to access the accuracy of both TFEA and AME.

**Fig. 4:**
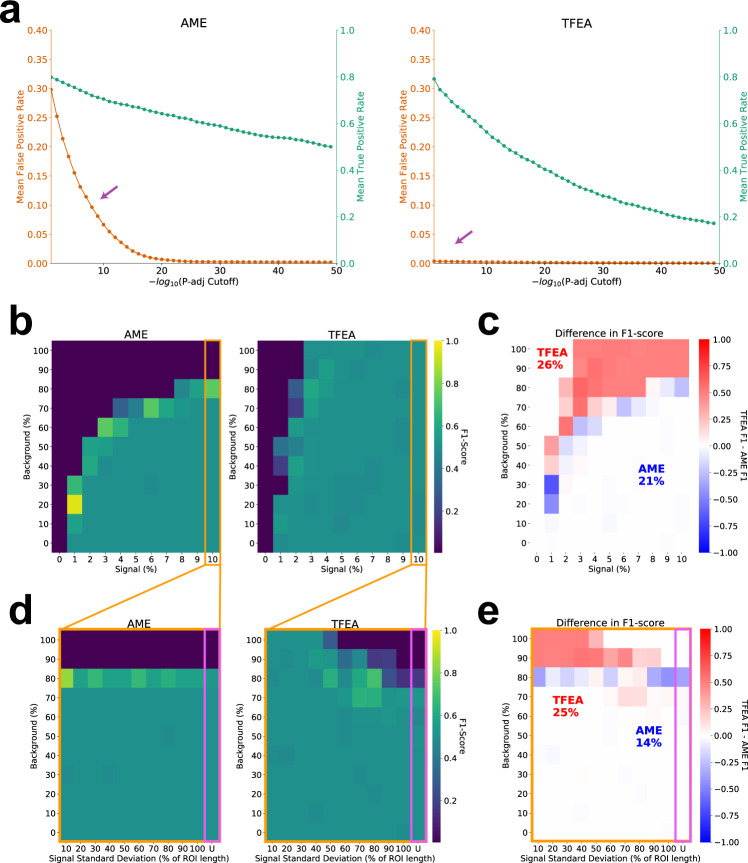
TFEA balances TF positional and differential signal. **a** Optimal cutoffs are determined using the mean true positive rate (TPR; green) and mean false positive rate (FPR; orange) across the different signal and background levels as a function of varying the threshold cutoff. **b** F1 score of AME and TFEA for varied signal and background, using optimal AME cutoff 1e−30 and TFEA cutoff 0.1. **c** Difference in F1 score between TFEA and AME across all simulations (*n* = 121; value = *F*1_TFEA_ − *F*1_AME_). TFEA (red) outperforms AME (blue) in 26% of cases (value > 0) whereas AME outperforms TFEA in 21% of cases (value < 0). **d** F1 scores and **e** difference in scores for highest signal tested (10% signal), now varying the standard deviation of the signal and background. See Supplementary Fig. 17 for more details on simulations.

We first measured the mean false-positive rate (FPR) and mean true positive rate (TPR) across tests of varying signal and background (Fig. 4a). We found that AME detected many false positives (defined as any motif besides *TP53*) at loose threshold cutoffs and therefore chose a strict cutoff of 1e−30 for AME. TFEA on the other hand, had a very low FPR even at loose thresholds with the TPR decreasing as the cutoff became stricter. We, therefore, chose a cutoff of 0.1 for TFEA.

We next generated two sets of simulated datasets to evaluate the performance of each method with varying signal/background (Fig. 4a) or variance/background (Fig. 4b). For each scenario, we generated ten simulations and measured F1 scores for AME and TFEA. Varying signal/background (Figure 4b), we found that at high background levels (above 80%), AME was no longer able to detect the enrichment of *TP53*. TFEA on the other hand was able to detect *TP53* even at high background levels by incorporating positional information. Computing the differential F1 scores between the two methods (Fig. 4c) shows that TFEA performs well in cases where AME detects no enrichment of *TP53* (26% of cases), whereas AME outperforms TFEA in 21% of cases. Importantly, because AME does not take positional information into account, it was never able to capture cases where the level of signal and background are similar.

To further determine how TFEA handles the loss of positional information, we chose the highest signal level tested and altered the variance (standard deviation) of the signal position and the background level (Fig. 4d). As expected, AME shows consistent behavior regardless of the positional information of the motif. In contrast, TFEA is able to distinguish signals with differing levels of positional localization. In the extreme case of no positional localization (motifs embedded with a uniform distribution), TFEA performs only slightly worse than AME (Fig. 4e).

Finally, we sought to benchmark the runtime performance and memory usage of TFEA against AME. Here we leverage a first-order Markov model (see Methods section “TFEA: Testing compute performance” to simulate increasing numbers of ROIs as input. Analyzing the core collection of HOCOMOCO TF motifs (*n* = 401), we found that AME runtime increased non-linearly while TFEA runtime increased linearly with a single processor (Supplementary Fig. 18a). Importantly, TFEA can utilize parallel processing, leading to notably faster runtimes. In terms of memory usage, although TFEA consumes more memory than AME, even in the worst case of 100,000 input regions, TFEA’s memory footprint is less than 1 Gb and therefore can still be run on a local desktop computer (Supplementary Fig. 18b).

### TFEA outperforms AME on experimental time-series data

We next sought to examine the performance of TFEA and AME on real biological data. Here, we utilized cap analysis of gene expression (CAGE), which precisely defines the TSS of individual transcripts^49–51^. We analyzed a CAGE-seq time-series dataset from the FANTOM consortium^50,52^. In this dataset, human-derived monocytes were differentiated into macrophages and treated with lipopolysaccharide (LPS), a proxy for bacterial infection. Differential expression analysis was performed on each LPS time point comparing treatment to control to obtain a list of ranked ROIs.

**Fig. 5:**
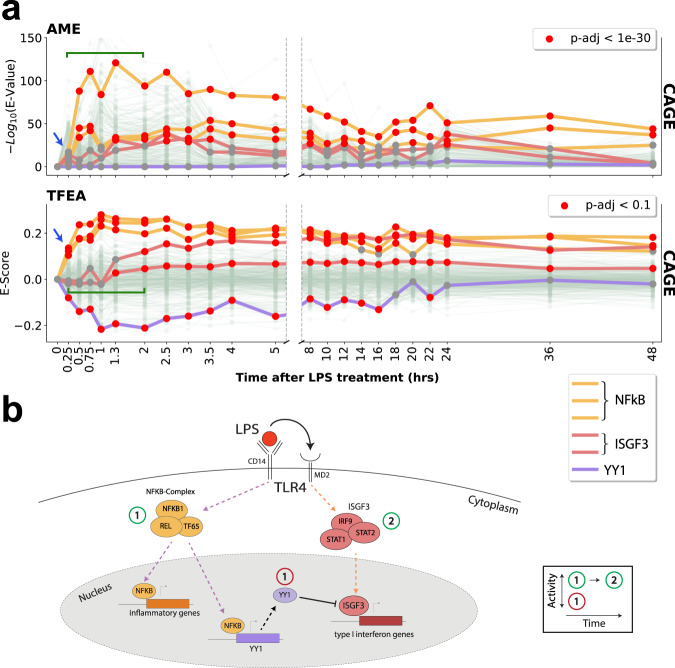
TFEA dissects the temporal dynamics of infection. **a** Analysis of lipopolysaccharide (LPS) time-series cap analysis gene expression (CAGE) data^10, 25^ using AME and TFEA. Trajectories of activity profiles show LPS triggers immediate activation of the NF-*κβ* complex (TF65/RelB/NFKB1; yellow), observable at 15 min (blue arrow). TFEA detects a concomitant downregulation of a set of transcription factors, exemplified here by TYY1 (purple). TFEA also resolves subsequent dynamics (green bracket) of ISGF3 activation (containing IRF9/STAT1/STAT2; red lines). **b** Schematic depicting the molecular insights gained from TFEA analysis. See Supplementary Fig. 19 for more analysis. See Supplementary Data 1 for a complete list of accession numbers for data utilized.

TFEA recovered the immediate innate immune response, exemplified by the most rapid reported (within 15 min) activation of NF-*κβ* (*RELA*, *RELB*, and *NFKB1*; Fig. 5a). In addition, TFEA temporally resolved the known secondary response that arises at later time points, which includes the activation of the IFN-stimulated gene factor 3 (ISGF3)^53^ complex, comprising *IRF9* and *STAT1*/*STAT2*^54^. In contrast, AME did not recover the innate immune response at the earliest time point and provided less temporal resolution when distinguishing primary and secondary responses.

Concurrent with the immediate innate immune response, TFEA identified a set of TFs that exhibit a rapid decrease in E-Scores including *ELF1*/*ELF2*^55^, *YY1*^56,57^, *USF1*/*USF2*^58^, and *GABPA*^59^. The decreased *E*-score set includes *YY1*, a transcriptional inhibitor known to be activated directly by NF*κ*B^60^. Reduction in the *E*-score of *YY1* illustrates an important limitation of TFEA—namely, that it cannot distinguish between the activation of a repressor or the loss of an activator. Ultimately, we show with this proof of principle that if the cellular response to LPS was not known a priori, we could temporally resolve key aspects of the regulatory network using TFEA and dense time series CAGE data (Fig. 5b and Supplementary Fig. 19).

### TFEA works on numerous regulatory data types that inform on RNA polymerase initiation

We developed *muMerge* and TFEA for the purpose of inferring TF activity from high-resolution data on transcription initiation, such as precision run-on sequencing (PRO-seq) or CAGE. However, numerous genomic datasets aimed at transcriptional regulation have a clear relationship with RNA polymerase initiation (see Methods section “Regions of interest”). For example, RNA polymerase initiation originates in open chromatin regions. Although these data sets are less precise and are not direct readouts of polymerase initiation, the popularity of these data makes them readily available. To determine whether TFEA could adequately infer TF activity from these datasets, we analyzed a time-series dataset from ENCODE^2,61^ in which cells were treated with dexamethasone (Dex)—a known activator of the glucocorticoid receptor (GR).

**Fig. 6:**
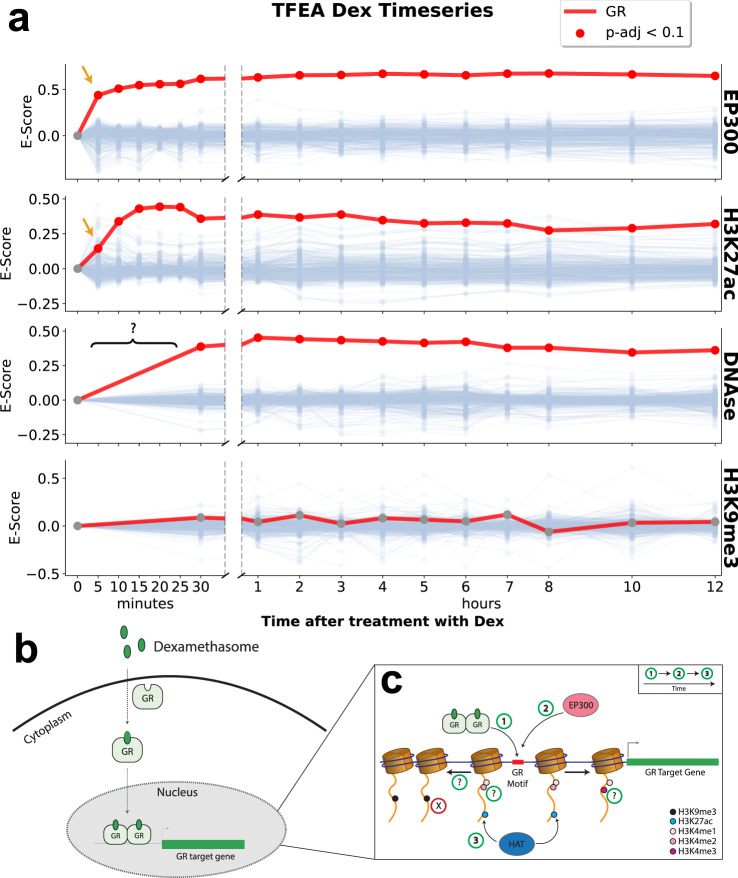
TFEA captures rapid dynamics of the glucocorticoid receptor (GR) following treatment with dexamethasone. **a** TFEA correctly identifies GR (red line) from time-series ChIP data on the histone acetyl-transferase p300, H3K27ac, and DNase I^61^. No signal is observed in the negative control H3K9me3. TFEA shows a temporal lag in the H3K27ac signal (orange arrows). **b** Known cellular dynamics of GR induced by dexamethasone (Dex). **c** Mechanistic and temporal insights gained by performing TFEA analysis, question marks indicate datasets where earlier time points were not available to resolve temporal information. See Supplementary Data 1 for a complete list of accession numbers for data utilized.

TFEA correctly identifies GR as the key responding TF from the datasets that most closely capture RNA polymerase initiation (including p300, H3K27ac, and DNA accessibility), and does not identify GR for the transcriptionally repressive mark H3K9me3 (Fig. 6a)^61,62^. Surprisingly, the effects of p300 and H3K27ac are seen rapidly, as soon as 5 min after dexamethasone treatment. Furthermore, H3K27ac deposition is temporally lagged behind its canonical acetyl-transferase p300^63–65^. In addition, the enhancer marks H3K4me1 and H3K4me2 show strong enrichment of GR by 30 min but the promoter mark H3K4me3 shows only modest enrichment, further supporting the finding that GR binds primarily at enhancers^61^ (Supplementary Fig. 20). Using the diversity of data types and dense time series, we can construct a temporally resolved mechanism of how GR affects changes in transcription (Fig. 6b, c).

## Discussion

We present here TFEA, a computational method that seamlessly balances the information obtained from differential transcription with the position of a nearby motif, thereby allowing it to be broadly applicable to a variety of datasets that approximate RNA polymerase initiation regions. We show that TFEA outperforms existing enrichment methods when positional data is available and is comparable to these methods in the absence of positional signals. Further, we show that TFEA, when leveraged with high-resolution time-series data, can provide mechanistic insight into the order of regulatory events responding to a perturbation.

A key aspect of TFEA is the incorporation of both positional and differential information in calculating TF motif enrichment. Most motif enrichment algorithms use solely differential information, likely due to the poor positional resolution of historically popular techniques such as ChIP-Seq. Methods such as nascent transcription and CAGE provide higher resolution on the position of RNA polymerase initiation genome-wide. To leverage the improved resolution of these methods, we introduce *muMerge*—a statistically principled way of combining ROIs across replicates and conditions that better captures position and length-scale information as compared to standard merging or intersecting approaches. The presence of improved positional information greatly increases the ability to detect biologically relevant TFs.

Although TFEA makes substantial improvements in detecting which TFs are associated with changes to RNA polymerase in response to perturbations, there are several aspects of this approach that could be improved. First, TFEA inherits some limitations from its dependence on both DESeq and a collection of motifs (see Methods section “Limitations to TFEA and muMerge” for more details). More integral to the enrichment metric, TFEA motif scanning currently requires a fixed cutoff. Future iterations of the method could conceivably eliminate this cutoff, but likely this will substantially increase run times for what may only be minor gains in performance. Finally, sites of transcription initiation (both promoters and enhancers) show substantial GC bias. While we made some effort to account for this bias using linear regression, a more principled approach is desired.

Despite these caveats, TFEA recovers known TF dynamics across a broad range of data types in response to a variety of perturbations. Inevitably, the data type utilized influences the detection ability of TFEA. For example, while CAGE data provides precise resolution on the TSS, it must be deeply sequenced to detect some enhancer-associated transcription events^31^. Consequently, TFs that predominantly regulate enhancers will likely be less detectable in poorly sequenced CAGE data. On the other hand, some methods are more capable of detecting immediate changes in RNA polymerase initiation, such as precision run-on sequencing, allowing for shorter, more refined time points. As demonstrated here, TFEA is able to leverage the information from each data set by incorporating both its distinct positional and differential signal. Applying TFEA to diverse data types, using dense time series, can uncover a detailed mechanistic understanding of the key regulators that enact the cell’s dynamic response to a perturbation.

## Methods

### TF enrichment analysis

We have developed TFEA to identify TFs that demonstrate significant differential activity following a perturbation. It has been observed that, during a perturbation, the binding sites of active TFs co-localize with regulatory regions that exhibit strong differential RNA polymerase initiation^26^. TFEA leverages this observation to calculate an enrichment score that quantifies the co-localization of TF motif instances with sites of altered RNA polymerase activity.

Here we describe in detail the key steps of the TFEA pipeline (shown in Fig. 1)—specifically, for each TF we describe how the main input (ROIs) are defined, how the ROIs are ranked, and how the enrichment score is subsequently calculated and GC-corrected.

#### Regions of Interest

One input required for TFEA is a common set of ROIs on which all experimental samples are evaluated. Each region (consisting of a genomic start and stop coordinate) represents a reference point (the midpoint of the region) and uncertainty on that reference point (the width of the region).

The biological interpretation of an ROI depends on the nature of the data type being used. However, it is assumed the data being used captures some aspect of RNA polymerase initiation (e.g., CAGE-, Pol II ChIP-, p300 ChIP-, nascent-, or ATAC-seq), to varying degrees of precision, depending on the assay. Specifically, using CAGE data provides a highly precise measurement of each TSS, thus the ROI would be narrow and centered on the TSS. With nascent transcription data, such as PRO-seq or GRO-seq, the position of RNA polymerase loading and initiation (e.g., the midpoint between two bidirectional TSS)^45^ is often identified^26,66^. RNA polymerase II ChIP also informs on the RNA polymerase loading and initiation region, but at lower resolution to nascent transcription data^31^. Likewise, H3K4me 1/2/3 has been shown to correlate with transcription levels^31^ but flank the site of initiation^45^. Finally, as nearly all sites of RNA polymerase loading and initiation originate within open chromatin regions, ATAC-seq data (and related accessibility metrics) are also informative^45^ but at lower positional precision and with more false positives (open regions without transcription)^67^.

Regardless of the assay, most methods identify such regions independently in each dataset (e.g., a peak caller for ChIP data or Tfit for identifying sites of bidirectional transcription in nascent data). As a result, these regions will not (and should not) be exactly consistent between samples (e.g., some sites are condition-specific and, even for shared sites, boundaries may vary). Therefore, a principled method is needed to combine the regions from all the samples into a consensus set.

#### Defining ROIs with *muMerge*

In order to combine regions from multiple samples into a consensus set of ROIs, we developed a probabilistic, principled method we call *muMerge*. Initially, *muMerge* was specifically developed for determining the set of consensus RNA polymerase loading and initiation sites observed in nascent sequencing data (by combining bidirectional calls from multiple samples) but it can be applied to peak calls generated from other regulatory data types as well (e.g., ChIP, ATAC, or histone marks).

The basic assumption made by *muMerge* is that each sample is an independent observation of an underlying set of hypothetical loci—where each hypothetical locus has a precise critical point *μ*, of which the corresponding sample region ([*start*, *stop*]) is an estimate. We assume the true coordinate of the locus is more likely to be located at the center of the sample region than at the edges, so *muMerge* represents the sample region by a standard normal probability distribution, centered on the region, whose standard deviation is related to the region width.

To calculate the best estimate (the ROI) for a given locus, *muMerge* calculates a joint probability distribution across all samples from all regions that are in the vicinity of the locus. This joint distribution is calculated by assuming

1. replicates within a condition are independent and identically distributed (*i.i.d*.)
2. replicates *across* conditions are mutually exclusive (i.e., a sample cannot represent multiple experimental conditions)

Hence *muMerge* computes the product of the normal distributions across all *replicates* within a condition and then sums these results across all *conditions*. The best estimates for the transcription loci *μ* (there may be multiple) are taken to be the local maxima of this joint distribution—these are the ROI positions. Finally, to determine an updated width, or confidence interval, for each ROI, *muMerge* assumes that the original sample regions whose midpoints are closest to the new position estimate are the most informative for the updated width. Thus the ROI width is calculated by a weighted sum of the widths of the original regions, weighted by the inverse of the distance to each one.

#### *muMerge* mathematical description

Principally, *muMerge* makes two probabilistic assumptions about sequence samples:

- **Assumption A:** Replicate samples are independent measurements of *identical experimental conditions* and therefore any corresponding sample regions within them are independent and identically distributed (*i.i.d*.) observations of a common random variable (*i.e*., the underlying hypothetical locus).
- **Assumption B:** Cross-condition samples are independent measurements of *mutually exclusive experimental conditions* and therefore any sample regions within them are observations of (potentially) disjoint random variables.

These two assumptions inform how *muMerge* accounts for each individual sample, when computing the most likely ROI for any given genomic location (see below for further details).

To start, the inputs to *muMerge* are a set of regions for each sample (genomic coordinates: {[*start*, *stop*], . . . }) that represent the sequenced features present in the dataset, as well as an experimental conditions table that indicates the sample groupings (which samples are from which experimental condition). With these inputs, *muMerge* performs the following steps to compute a global set of ROIs:

1. Group overlapping sample regions (each group is processed one at a time)
2. Express each sample region as a positional probability distribution (Eq. (1))
3. Generate a joint distribution (Eq. (2))
4. Identify local, maximum likelihood ROI positions from the joint distribution
5. Compute ROI widths via weighted sum (Eq. (3))
6. Adjust the sizes of overlapping ROIs
7. Record final ROIs for the given group
8. Repeat 2–7 for all remaining groups

Now we describe these steps in detail: First, from the input samples, *muMerge* groups all sample regions that overlap in genomic coordinate (a region is grouped with all other regions it overlaps and, transitively, with any regions overlapping those). We denote a single group of overlapping regions as *G*_*r*_. This grouping is done globally for all samples, resulting in a set of grouped regions *G* = {*G*_*r*_}, such that every sample region is contained in exactly one grouping *G*_*r*_ (i.e., ${G}_{r}\cap {G}_{s}={{\emptyset}},\ \forall \ r{\,}\ne{\,} s$) (step 1). Then each group of regions, *G*_*r*_, is processed individually, as the remainder of this section describes (steps 2–7). For a given group, we denote each sample region within it as the 2-tuple ${({\mu }_{k},{\sigma }_{k})}_{ij}\in {G}_{r}$, where *μ*_*k*_ is the genomic coordinate (base position) of the center of the region and *σ*_*k*_ is the region half-width (number of bases) (shown schematically in Supplementary Fig. 3a “Sample Regions”). In the 2-tuple, the indices denote the *k*th sample region for replicate *j* in condition *i*.

*muMerge* then processes the regions in *G*_*r*_ as follows. Each region within the group is expressed as a standard normal distribution (*ϕ*) as a function of base position *x*

1 $${({\mu }_{k},{\sigma }_{k})}_{ij}\to {p}_{ij}^{(k)}(x)=\phi \left(\frac{x-{\mu }_{k}}{\rho \ {\sigma }_{k}}\right)$$

where *ρ* is the “width ratio”—the ratio of the half-width sample region to the standard deviation of the normal distribution—with a default of *ρ* = 1 (user option) (shown schematically in Fig. 2a and Supplementary Fig. 3b “Generate probability distribution”). This distribution represents the probability of the location for the underlying hypothetical locus (*μ*), which ${({\mu }_{k},{\sigma }_{k})}_{ij}$ is an estimate. For those samples with no regions within *G*_*r*_, the probability distribution is expressed as uniform, ${p}_{ij}^{(k)}(x)=1/{{\Delta }}$ where Δ is the full range encompassed by the overlapping sample regions. In other words, we assume that if the sample contains no data to inform the location of the underlying loci at that location, then all positions are equally likely for that sample. *muMerge* then calculates a joint distribution (${{\mathcal{P}}}_{\rm{joint}}(x\ | \ {p}_{ij})$) by combining all ${p}_{ij}^{(k)}(x)$ for the group as follows:

2 $${{\mathcal{P}}}_{\rm{joint}}(x\ | \ {p}_{ij})=\mathop{\sum}\limits_{i}\left(\mathop{\prod}\limits_{j}\left(\mathop{\sum}\limits_{k}{p}_{ij}^{(k)}(x)\right)\right)$$

Here we are calculating the product of the replicate distributions (index *j*—those within a given experimental condition), consistent with our probabilistic assumption A, and the sum of the resulting distributions across experimental conditions (*i* index), consistent with our probabilistic assumption B (shown schematically in Fig. 2a and Supplementary Fig. 3c “Calculate joint probability”). Examples of how ${{\mathcal{P}}}_{\rm{joint}}$ would be calculated for a given experimental set-up are given in Supplementary Figure 21. Though this function is not a normalized probability distribution, we are only interested in relative values of ${{\mathcal{P}}}_{\rm{joint}}(x\ | \ {p}_{ij})$. Specifically, we are interested in the maxima of this function. We identify the set of maxima (which we denote $\{{\widehat{\mu }}_{k}\}$) and rank them by the function value for each position, ${{\mathcal{P}}}_{\rm{joint}}(x={\widehat{\mu }}_{k}\ | \ {p}_{ij})$. We then keep the top *M* + 1 from the ranked set, where *M* is the median number of regions per sample in *G*_*r*_ (user option). This is our final set of estimates on the hypothetical loci positions, *μ*—i.e., the positions of our ROIs for group *G*_*r*_.

For each ${\widehat{\mu }}_{k}$, we then calculate a width for the resulting ROI. We do so for each by calculating a weighted sum over the set of all original sample regions in the group, $\{{({\mu }_{k},{\sigma }_{k})}_{ij}\}$, weighted by the inverse of the distance from the final position estimate to each *μ*_*k*_ (shown in Supplementary Fig. 3d “width estimation”). Thus the final ROI half-width, ${\widehat{\sigma }}_{k}$, is calculated as follows:

3 $${\widehat{\sigma }}_{k}=\mathop{\sum}\limits_{i}\frac{{\sigma }_{i}}{| {\widehat{\mu }}_{k}-{\mu }_{i}| +1}/\mathop{\sum}\limits_{i}\frac{1}{| {\widehat{\mu }}_{k}-{\mu }_{i}| +1}$$

where *i* indexes all sample regions in the group ${G}_{r}=\{{({\mu }_{k},{\sigma }_{k})}_{ij}\}$. Our rationale is that the width of those sample regions that are closer to the ROI position ${\widehat{\mu }}_{k}$, are more informative for the ROI width and therefore are given a larger weight. This results in a set of ROIs $\{({\widehat{\mu }}_{k}-{\widehat{\sigma }}_{k},\ {\widehat{\mu }}_{k}+{\widehat{\sigma }}_{k})\}$ (shown in Supplementary Fig. 3e “Final region estimate”).

Finally, we determine if there is overlap between any of the regions in this set of ROIs. If so, any two overlapping regions are reduced in size, symmetrically about their centers, until they no longer overlap. This is done so that any genomic position can be uniquely associated with an ROI. The final ROIs for the group are then written to an output file to be used downstream in the pipeline. This process is repeated for all groups of overlapping sample regions (i.e., ∀ *G*_*r*_ ∈ *G*).

#### Ranking ROIs

With a set of ROIs identified, the next step is to rank them by differential signal. Because the goal of TFEA is to identify TFs that are responding to a perturbation, a ranking based on the differential transcription at the ROIs would capture the regulatory behavior of the TF. Technically, the signal in each data type actually represents different biological processes—differential transcription for nascent (PRO-seq or GRO-seq), differential accessibility (DNAse or ATAC-Seq), and differential occupancy for ChIP. Logically, we assume each is a reasonable proxy for differential transcription. There are a number of ranking metrics one could use that are based on these differential signals—for example, the difference in coverage, log-fold change, or a differential significance (*p* value). For TFEA, we chose to rely on a well-established tool (DESeq) to perform our ranking, since it was designed to model the statistical variation found in sequencing data^43^.

For a set of ROIs, TFEA calculates read coverage for each replicate and condition using *bedtools multibamcov* (version 2.25.0)^68^. TFEA then inputs the generated counts table into DESeq2 (v 1.26)^43^ (or DESeq (v 1.38)^42^ if no replicates are provided) to obtain differential read coverage for all ROIs. By default, these regions are then ranked by the DESeq computed p-value, separated by positive or negative log-fold change (alternative user option to rank the ROIs purely by fold-change). In other words, the ROIs are ranked from the most significant positive fold-change to the most significant negative fold-change.

#### Identifying locations of motif instances

Accurately identifying the locations of motif instances relative to each ROI is a critical step in the TFEA pipeline. By default, TFEA uses the motif scanning method FIMO, which is a part of the MEME suite (version 5.0.3)^69^. FIMO represents each TF by a base-frequency matrix and uses a zero-order background model to score each position of the input sequences. For each ROI, we scan the 3kb sequence surrounding the ROI center (${\widehat{\mu }}_{i}\pm 1.5\,{\rm{kb}}$). This 3kb window was chosen primarily to reduce computation time and is also consistent with the window used for the MD-Score method^26^. For each TF, we utilize a scoring threshold of 10^−6^ and keep the highest scoring position (denoted *m*_*i*_), in the event more than one motif instance is identified. If no position score above the threshold, then no *m*_*i*_ is recorded for the ROI. Our background model is determined by calculating the average base frequency overall ROI. For this paper, we use the frequency matrices from the HOCOMOCO database^48^ with a default pseudo-count of 0.1.

#### Enrichment score

With the motif instances identified for each of the ranked ROIs, we now detail how TFEA calculates the enrichment score (“E-score”—in Fig. 1) for each TF. The procedure for calculating enrichment requires two inputs:

1. N-tuple ordered list $({\widehat{\mu }}_{i})$—the genomic coordinates for reference points, assumed to be the centers of all ROIs (*e.g*., consensus ROIs calculated by *muMerge*), ranked by DESeq *p* value (separated by the sign of the fold-change).
2. Ordered list (*m*_*i*_)—the genomic coordinates of each max-scoring motif instance (e.g., motif locations generated by scanning with FIMO), for each ROI.

We first calculate the motif distance *d*_*i*_ for each ROI—the distance from each ${\widehat{\mu }}_{i}$ to the highest scoring motif instance *m*_*i*_ within 1.5 kb of ${\widehat{\mu }}_{i}$. If no *m*_*i*_ exists within 1.5 kb, then *d*_*i*_ is assigned a null value ($\oslash$) (Eq. (4)).

4 $${d}_{i}=\left\{\begin{array}{ll} | {\widehat{\mu }}_{i} - {m}_{i} | , &\, {\text{if}}\, {m}_{i} \,{\text{is}} \, {\text{present}}\hfill \\ {\oslash},\hfill &{\text{if}}\, {m}_{i} \,{\text{is}}\,{\text{not}}\,{\text{present}}\end{array}\right.$$

We use the distribution of these distances to calculate a weighted contribution to the E-score for each motif instance. In previous work, it has been observed that the distribution of motif position relative to sites of RNA polymerase initiation decays rapidly with increased distance^26^. Thus we have chosen to model the motif weights with an exponential function, whose decay length is independently determined for each TF, from the background motif distribution. In order to compute the weight model, we next calculate the background distribution of motif distances. We assume the majority of the ROIs experience no significant fold-change—namely, those ROIs in the middle of the ranked list. Consequently, we calculate the mean, background motif distance (Eq. (5)) for those ROIs whose rank is between the first and third quartiles of the ordered list of ROI positions, $({\widehat{\mu }}_{i})$, as follows

5 $${\bar{d}} = {\rm{mean}}\{ {d}_{i}\, | \, \forall \, i,\, {\text{if}}\, {Q}_{1} \le i \le {Q}_{3} \,{\text{and}}\, {d}_{i}\, \ne \, \oslash \}$$

where *Q*_1_ and *Q*_3_ are the first and third quartiles, respectively. Our assumption is that the interquartile range of the ordered list $({\widehat{\mu }}_{i})$—between indices *Q*_1_ and *Q*_3_—represents the background distribution of motif distances for the given TF, and therefore defines the weighting scale for significant ROIs in our enrichment calculation. We found this to be essential since the background distribution varies between TFs. This variation in the background can be attributed to the random similarity of a given motif to the base content surrounding the center of ROIs. For example, in the case of RNA polymerase loading regions identified in nascent transcription data (which demonstrate a greater GC-content proximal to *μ* as compared to genomic background^26^), GC-rich TF motifs were more likely to be found proximal to each ROI by chance and thus resulted in a smaller $\bar{d}$ than would be the case for a non-GC-rich motif.

Having calculated the mean background motif distance, we proceed to calculate the enrichment contribution (i.e., weight—Eq. (6)) for each ROI in the ordered list (see “Weight Calculation” in Fig. 1).

6 $${w}_{i} = \left\{\begin{array}{ll}{e}^{{-{{d}_{i}}/{\bar{d}}}},&\,{\text{if}}\, {d}_{i}\; \ne\; \oslash \hfill\\ 0,\hfill &\,{\text{if}}\, {d}_{i} \, = \, \oslash \end{array}\right.$$

In order to calculate the E-score, we first generate the enrichment curve for the given TF (solid line in “Enrichment Curve” in Fig. 1) and the background (uniform) enrichment curve (dashed line in “Enrichment Curve” in Fig. 1). We define the *E*-score as the integrated difference between these two (scaled by a factor of 2, for the purpose of normalization). The enrichment curve (Eq. (7)), which is the normalized running sum of the ROI weights, and the *E*-score (Eq. (8)) are calculated as follows:

7 $$e(i)=\frac{\mathop{\sum }\nolimits_{k = 0}^{i}{w}_{k}}{\mathop{\sum }\nolimits_{k = 0}^{N}{w}_{k}}$$

8 $$E=\frac{2}{N}\mathop{\sum}\limits_{i}\left(e(i)-\frac{i}{N}\right)$$

where *i* is the index for the ROI rank and *i*/*N* represents the uniform, background enrichment value for the *i*th of *N* ROIs. The background enrichment assumes every ROI contributes an equal weight *w*_*i*_, regardless of its ranking position. Therefore, the enrichment curve (Eq. (7)) will deviate significantly from the background if there is a correlation between the weight and ranked position of the ROIs. In this case, the *E*-score will significantly deviate from zero, with *E* > 0 indicating either the increased activity of an activator TF or decreased activity of a repressor TF. Likewise, *E* < 0 indicates either a decrease in an activator TF or an increase in a repressor TF. By definition, the range of the *E*-score is −1 to +1.

Unlike GSEA, which uses a Kolmogorov–Smirnov-like statistic to calculate its enrichment score^44^, the TFEA *E*-score is an area-based statistic. GSEA was designed to identify if a predetermined, biologically related subset of genes is over-represented at the extremes of a ranked gene list. Therefore, the KS-like statistic is a logical choice for measuring how closely clustered are the elements of the subset since it directly measures the point of greatest clustering and otherwise is insensitive to the ordering of the remaining elements. Conversely, because TFEA’s ranked list does not contain two categories of elements (the ROIs) and all elements can contribute to the E-Score, we wanted a statistic that was sensitive to how all ROI in the list were ranked—for this reason, we chose the area-based statistic. The null hypothesis for TFEA assumes all ROIs contribute equally to enrichment, regardless of their motif co-localization and rank. Hence the uniform background curve, to which the enrichment curve is compared.

In order to determine if the calculated *E*-score (Eq. (8)) for a given TF is significant, we generate an *E*-score null distribution from random permutations of $({\widehat{\mu }}_{i})$. We generate a set of 1000 null *E*-scores $\{{E}_{i}^{\prime}\}$, each calculated from an independent random permutation of the ranked ROIs, $({\widehat{\mu }}_{i})$. Our *E*-score statistic is zero-centered and symmetric, therefore we assume $\{{E}_{i}^{\prime}\} \sim {\mathcal{N}}({E}_{0},{\sigma }_{E}^{2})$. The final *E*-score for the TF is compared to this null distribution to determine the significance of the enrichment.

Prior to calculating the *E*-score *p* value, we apply a correction to the *E*-score based on the GC-content of the motif relative to that of all other motifs to be tested (user-configurable). This correction was derived based on the observation that motifs at the extremes of the GC-content spectra were more likely to call significant across a variety of perturbations. We calculate the E-Scores for the full set of TFs as well as the GC-content of each motif, {(*g*_*i*_, *E*_*i*_)}. We then calculate a simple linear regression for the relationship between the two

9 $$\widehat{b}=\bar{E}-\widehat{m}\bar{g}$$

10 $$\widehat{m}=\frac{\mathop{\sum }\nolimits_{i = 1}^{n}({g}_{i}-\bar{g})({E}_{i}-\bar{E})}{\mathop{\sum }\nolimits_{i = 1}^{n}({g}_{i}-\bar{g})}$$

11 $${E}_{GC}(g)=\widehat{b}+\widehat{m}g$$

where $\bar{E}$ and $\bar{g}$ are the average *E*-score and average GC-content. *E*_*GC*_(*g*) is the amount of the *E*-score attributed to the GC-bias for a motif with GC-content *g*. Thus the final *E*-score for the TF is given by *E*_TF_ = *E* − *E*_GC_(*g*_TF_), the difference between Eqs. (8) and (10). If GC-content correction is not performed, then Eq. (8) is taken to be the final *E*-score. The *p* value for the final TF *E*-score is then calculated from the *Z*-score, *Z*_TF_ = (*E*_TF_ − *E*_0_)/*σ*_*E*_.

### Limitations to TFEA and *muMerge*

Though *muMerge* and TFEA clearly demonstrate good performance, there are a number of limitations to both tools. We want to bring attention to these limitations so that users may better understand how best to apply these tools to data and interpret the results.

As implemented, *muMerge* assumes every input data set is of equal quality, by default. This means every data set is given equal weight when computing the joint probability. However, if some data sets are of low or questionable quality such that they have inaccurate bed regions, this may bias the ROI inferred by *muMerge*. We recommend removing poor quality data sets from those input to *muMerge* or weighting each data set based on its perceived quality. In short, *muMerge* cannot substitute for thoughtful quality control of each of one’s samples.

Additionally, sites with regions that are very closely spaced tend to be inferred as a single ROI by *muMerge*. This can be accounted for somewhat by decreasing the value of the width ratio (*ρ*, default value 1), which reflects the assumed uncertainty on the location of the input sample regions. However, there is no definitive ground truth value. It should be noted, both of these limitations also apply to the *bedtools* methods of combining regions.

As implemented, TFEA depends on DESeq to order the observed transcription changes between conditions. Consequently, TFEA performs best when replicates are available. Likewise, when DESeq assumptions are violated, this can result in unreliable region ordering. For example, when TF ChIP is utilized across conditions there are often large gains in binding events. This is particularly true with environmentally stimulated TFs such as p53 or GR which can be activated by Nutlin-3a and dexamethazone, respectively. The pre-stimulated condition typically has few (if any) detectable binding sites whereas post stimulation binding is detected at hundreds to thousands of sites. In this scenario, the DESeq assumption that the bulk of sites are unchanged is violated. Even if an alternative method of ordering sites were utilized, most gained sites contain the TF motif, so no enrichment is typically observed. For this reason, we do not recommend applying TFEA to environmentally responsive TF ChIP data sets or any other data type that clearly violates the statistical assumptions of DESeq.

Additionally, TFEA depends on a collection of known motifs. Unfortunately, some TFs have no known motif or one of poor quality. However, over time, the quality and numbers of TFs in the major databases have dramatically improved^3^. Furthermore, TFEA can only distinguish between paralogous TFs to the extent that they have distinct motifs. Sites of transcription initiation (both promoters and enhancers) show substantial GC bias. Consequently, short high GC content motifs, which are exceedingly common in ROIs, sometimes appear to show significant changes with a perturbation. The extent to which these signals represent a biological process or a statistical anomaly is unclear.

Finally, TFEA identifies when the TF motif associates with sites of changing RNA polymerase initiation. By ordering the differential transcription signal by the direction of change, TFEA can determine whether the identified TF is associated with a transcription gain or loss. Prior work has shown that stimulation of an activator gives rise to increased eRNA activity nearby^21–23^, but loss of a repressor also leads to proximal increased eRNA activity^70^. Consequently, if a motif associates with transcriptional gain it may arise from either activation or repression of the TF.

### Benchmarking

In order to benchmark the performance of *muMerge* and TFEA, we performed a number of simulations that isolate the different parameters of *muMerge* and TFEA, comparing the performance to that of some commonly used alternatives. We ran these alternatives: AME 5.0.5 and bedtools version 2.28.0 (*merge and intersect*) using default parameters. Here we describe how the data for each test was generated.

#### *muMerge*: Simulating replicates for calculation of ROIs

To test the performance of *muMerge* in a principled manner, we first generate replicate data in a way that simulates the uncertainty present in individual samples. For each replicate, we perform 10,000 simulations of sample regions for a single locus and calculate the average performance. For each simulation, we assume a precise position and width for the hypothetical locus and model the uncertainty of each sample region with a binomial and Poisson distribution, respectively. The position of each sample region, *μ*_*i*_, is pulled from a symmetric binomial distribution *μ*_*i*_ ~ *B*(*n* = 100, *p* = 0.5), centered at zero. The half-width of each sample region, *σ*_*i*_, is pulled from a Poisson distribution *σ*_*i*_ ~ *Pois*(*λ* = 100). The specific distributions utilized to generate the sample regions are as follows:

12 $${\text{locus}}\,{\text{estimate}}\,\equiv \left\{\begin{array}{ll}\,{\text{position:}}\,\hfill &{\mu }_{i} \sim \mu +B(n=100,p=0.5)-np\\ \,{\text{half-width:}}\,&{\sigma }_{i} \sim Pois(\lambda =100)\hfill\end{array}\right.$$

Here *B*( ⋅ ) is the binomial distribution centered at *np* with success probability 0.5 and variance *np*(1 − *p*) = 25. Thus, the position estimator *μ*_*i*_ for a single sample region is centered at *μ*. *Pois*( ⋅ ) is the Poisson distribution, thus, the half-width for each sample region have mean and variance of *λ* = 100.

The first test (Supplementary Fig. 4a) consisted of inferring a single locus (located at *μ* = 0) from an increasing number of replicates. A sample region for each replicate was generated from Eq. (12). This simulation was repeated 10,000 times for each number of replicates being combined. The methods *muMerge*, *bedtools merge*, and *bedtools intersect* were applied to each of the 10,000 simulations. The average error on the midpoint (its deviation from the true locus position, *μ* = 0) and region width were calculated for the regions generated from each method, averaged over all 10,000 simulations. The behavior of the average positional error and region width as a function of number of combined replicates is shown in Fig. 2b (Test 1).

The second test (Supplementary Fig. 4b) consisted of inferring two loci (*μ*_1_ = − *x* and *μ*_2_ = + *x*) as the distance between those loci was increased (from *x* = 0 to 200). This simulation was repeated 10,000 times for each value of *x* (with 3 replicates). The distribution of the inferred positions and widths were plotted, using *muMerge*, *bedtools merge* and *bedtools intersect*. The distribution of positions and widths as a function of the distance between *μ*_1_ and *μ*_2_ are shown in Fig. 2c (Test 2).

#### TFEA: simulated motif enrichment

To generate test sequences for understanding the contribution of positional signal to motif enrichment, we randomly sampled 10,000 sequences from detected bidirectional in untreated HCT116 cells^22^. As this collection of ROI was obtained from nascent transcription data, it maintains true biological sequence signals. To simulate differential transcription, we randomly ordered the set of ROI. We then embedded instances of the TP53 motif in the highest-ranked sequences with a normal distribution with *μ* = 0 and *σ* = 150 (representative of signal). Importantly—p53 is known to NOT be activated in HCT116 DMSO samples^22^. To simulate background noise, we embedded instances of the TP53 motif with a uniform distribution to a percentage of the remaining sequences (chosen randomly). To calculate an F1 score, for each scenario of varying signal to the background we generated ten simulations. We then calculated the harmonic mean of precision and recall with the aggregate *p* values of all 10 simulations measuring all 401 TF motifs within the HOCOMOCO database (total 4010 TF motifs). True positives, in this case, were the 10 instances of the TP53 motif that should be significantly enriched. Any other significantly enriched TF motifs were considered false positives. We performed two sets of tests: (1) varying the amount of motif signal relative to the amount of background and (2) varying the standard deviation of motif position in the highest signal tested (10% signal; with the last scenario being uniform signal distribution) and the amount of background.

#### TFEA: testing compute performance

The base (ATGC) content of regulatory regions was calculated from the sites of RNA polymerase initiation inferred in HCT116 DMSO (using Tfit; described in ref. ^26^). One million 3kb sequences were subsequently generated based on the empirical probability of the positional base composition. We then randomly sampled (without replacement) an increasing number of sequences (up to 100,000) to be used in the computational processing tests. Run time and compute resources were measured using the Linux *time* command on a single node of a 70-node mixed-platform high-memory compute cluster running CentOS 7.4. To compute the runtime for a single processor, we added the *systime* and *usertime*. To compute memory usage for a single processor, we reran TFEA using only a single processor.

### PRO-Seq in MCF10A

We generated PRO-seq libraries for MCF10A cells before (DMSO) and 1 hour after Nutlin-3a, described in detail below. Our MCF10A cells carried a WTp53 insertion at the p53 locus, as they were developed for a study of p53 isoforms^71^. A complete description of the cell line construction is provided here for completeness.

*Cas9RNP formation*. sgRNA was formed by adding tracrRNA (IDT cat# 1072533) and crRNA (TP53 exon 2, positive strand, AGG PAM site, sequence: GATCCACTCACAGTTTCCAT) in a 1:1 molecular ratio together and then heating to 95 °C and then allowing to slowly cool to room temperature over 1 h. Cas9RNP was then formed by adding purified Cas9 protein to sgRNA at a ratio of 1:1.2. 3.7 μL of purified Cas9 protein at 32.4 μM was added to 2.9 μL of 50 μM sgRNA. This was then incubated at 37 °C for 15 min, and used at 10 μM concentration within the hour.

*Donor plasmid construction*. Vector Builder was used to constructing plasmid. The insert was flanked by 1.5 kb homology arms, and mCherry was inserted as a selection marker.

*CRISPR/Cas9 genome editing*. MCF10A cells cultured in DMEM/F12 (Invitrogen #11330-032) media containing 5% horse serum (LifeTech #16050-122), 20 ng/mL EGF ((Peprotech #AF-100-15), 0.5 μg/mL Hydrocortisone (Sigma #H0888-1g), 100 ng/mL Cholera toxin (Sigma #C8052-2 mg), 10 μg/mL insulin (Sigma #I1882-200mg), and 1× Gibco 100× Antibiotic–Antimycotic (Fisher Sci, 15240062) penicillin–streptomycin. Cells were split 24 h prior to the experiment and grown to approximately 70% confluency on a 15 cm plate. Media was aspirated, and the cells were washed with PBS. Totally, 4 ml of trypsin per plate were used to harvest adherent cells, after which 8 mL of resuspension medium (DMEM/F12 containing 20% horse serum and 1× pen/strep) was added to each plate to neutralize the trypsin. Cells were collected in a 15 ml centrifuge tube and spun down at 1000 × *g* for 5 min, then washed in PBS and spun down again at 1000 × *g* for 5 min. Cells were counted using a hemocytometer and 5 × 10^5^ cells were put in individual 1.5 mL Eppendorf tubes for transfection. Cells were re-suspended in 4.15 μL Buffer R, 10 μM Cas9RNP (6.6 μL), 1 μg WTp53 donor plasmid (1.25 μL). The mixture was drawn up into a 10 μL Neon pipet tip, electroporated using the Neon Transfection Kit with 10 μL tips (1400 V, 20 ms width, 2 pulses). Transfected cells were then pipetted into 2 mL of antibiotic-free media. After 1 week of recovery, cells were then single-cell sorted into 96-well plate based on mCherry expression. Clones were then verified with sequencing, PCR, and western blot.

*Replicates*. A single validated clone of MCF10A WTp53 cells was selected for subsequent PRO-seq analysis. All experiments were conducted in duplicate from separate cell growths.

*Nuclei preparation*. MCF10A WTp53 cells were seeded on three 25 cm dishes (1 × 10^7^ cells per dish) for each treatment 24 h prior to the experiments (70% confluency at the time of the experiment). Cells were treated simultaneously with 10 μM Nutlin-3a or 0.1% DMSO for 1 h. After treatment, cells were washed 3× with ice-cold phosphate-buffered saline, and then treated with 10 ml (per 15 cm plate) ice-cold lysis buffer (10 mM Tris-HCl pH 7.4, 2 mM MgCl_2_, 3 mM CaCl_2_, 0.5% NP-40, 10% glycerol, 1 mM DTT, 1× Protease Inhibitors (1 mM Benzamidine (Sigma B6506-100G), 1 mM Sodium Metabisulfite (Sigma 255556-100G), 0.25 mM Phenylmethylsulfonyl Fluoride (American Bioanalytical AB01620), and 4 U/mL SUPERase-In). Cells were centrifuged with a fixed-angle rotor at 1000 × *g* for 15 min at 4 °C. The supernatant was removed and the pellet was resuspended in 1.5 mL lysis buffer to a homogenous mixture by pipetting 20–30× before adding another 8.5 mL lysis buffer. The suspension was centrifuged with a fixed-angle rotor at 1000 × *g* for 15 min at 4 °C. The supernatant was removed and the pellet was resuspended in 1 mL of lysis buffer and transferred to a 1.7 mL pre-lubricated tube (Costar cat. No. 3207). Suspensions were then pelleted in a microcentrifuge at 1000 × *g* for 5 min at 4 °C. Next, the supernatant was removed and pellets were resuspended in 500 μL of freezing buffer (50 mM Tris pH 8.3, 40% glycerol, 5 mM MgCl_2_, 0.1 mM EDTA, 4 U/ml SUPERase-In). Nuclei were centrifuged 2000 × *g* for 2 min at 4 °C. Pellets were resuspended in 100 μL freezing buffer. To determine the concentration, nuclei were counted from 1 μL of suspension, and freezing buffer was added to generate 100 μL aliquots of 10 × 10^6^ nuclei. Aliquots were flash-frozen in liquid nitrogen and stored at −80 °C.

*Nuclear run-on and RNA preparation*. Nuclear run-on experiments were performed as described^72^ with the following modifications: the final concentration of non-biotinylated CTP was raised from 0.25 to 25 μM, a clean-up and size selection was performed using 1× AMPure XP beads (1:1 ratio) (Beckman) prior to test PCR and final PCR amplification, and the final library clean-up and size selection was accomplished using 1× AMPure XP beads (1:1 ratio) (Beckman).

*Sequencing*. Sequencing of PRO-Seq libraries was performed at the BioFrontiers Sequencing Facility (UC-Boulder). Single-end fragment libraries (75 bp) were sequenced on the Illumina NextSeq 500 platform (RTA version: 2.4.11, Instrument ID: NB501447), demultiplexed and converted BCL to fastq format using bcl2fastq (bcl2fastq v2.20.0.422); sequencing data quality was assessed using FASTQC (v0.11.5) and FastQ Screen (v0.11.0), both obtained from https://www.bioinformatics.babraham.ac.uk/projects/. Trimming and filtering of low-quality reads was performed using BBDUK from BBTools (v37.99) (Bushnell B. BBMap. http://sourceforge.net/projects/bbmap/) and FASTQ-MCF from EAUtils (v1.05)^73^.

#### Data processing

*p53 ChIP data*. Raw ChIP-seq data (GSE86222) from Andrysik et al.^40^ was downloaded from the SRA database. Data were processed with the ChIP-Flow pipeline (https://github.com/Dowell-Lab/ChIP-Flow) as follows. Reads were trimmed using BBduk from BBMap version 38.05 with the following flags ktrim=r qtrim=10 k=23 mink=11 hdist=1 maq=10 minlen=20^74^. Trimmed reads were mapped to the human reference genome (GRCh38/hg38) using HISAT2 version 2.1.0 with the –very-sensitive and –no-spliced-alignment flags^75^. Next, SAMtools version 1.8^76^ was used to convert sam files to sorted bam files, and duplicate reads were removed with Picard Tools version 2.6.0^77^. Finally, MACS2 version 2.1.1 was used to call peaks using each of the input samples for each cell line as control^78^.

*ENCODE data*. Raw bed and bam files were downloaded directly from ENCODE (encodeproject.org). These files were input directly into the *muMerge* or TFEA pipeline for processing and analysis. AME analysis was performed on the ranked ROI list produced as an optional output from TFEA.

*muMerge TF ChIP-seq comparison*. Peak calls for each region were scanned for an instance of the TF motif (from HOCOMOCO) using FIMO (MEME version 5.1.1), and peaks with significant hits to the TF motif (p-adj < 0.001) were retained^69^. Sample regions were combined across replicates (cell types) and conditions (with or without Nutlin-3a) with *muMerge*, *bedtools merge*, and *bedtools intersect* (bedtools version 2.28.0)^68^. The point of interest for *muMerge* was the center of the called peak, which was expanded by 1500 bp to specify the full ROI. Distance to the motif instance was calculated using the region midpoint compared to the midpoint of the best motif instance. For each method, we report the standard deviation, mean, and median of distances for each region.

*GRO/PRO-Seq data*. All GRO-Seq and PRO-Seq data were processed using the Nextflow^79^ NascentFlow pipeline (v1.1^80^) specifying the “–tfit” flag. Subsequent Tfit bed files from all samples were combined with *muMerge* to obtain a consensus list of ROIs.

*FANTOM data*. Raw expression tables for the Macrophage LPS time series were downloaded using the table extraction tool from the FANTOM Semantic catalog of Samples, Transcription initiation, And Regulations (SSTAR; http://fantom.gsc.riken.jp/5/sstar/Macrophage_response_to_LPS). Because the annotations for regions within hg38 counts tables contained “hg19”, we performed this analysis in the hg19 genome with the hg19 counts table instead of the hg38 counts table. We then performed DESeq analysis (since there were no replicates) on each time point compared to control and ranked the annotated regions within the counts table similar to Fig. 1. We then ran TFEA and AME with default settings on each of the three donors. We displayed only data for donor 2, as this sample had the most complete time-series data.

*Clustering FANTOM data*. We retained TFs with at least 15 significant (*p*-adj < 0.1) time points (representing 2/3 of all timepoints) from the TFEA output and applied K-means clustering. Clustering of the time series data was performed on the first two hours only, in order to distinguish the early responses to LPS infection. K-means clustering was conducted using the Hartigan and Wong algorithm with 25 random starts and 10 iterations for *k* = 3^81^. The optimal number of clusters was selected using the Elbow method^82^.

*String database analysis*. Protein names from TFs that were found to be significant in at least 15-time points were taken from the HOCOMOCO database. These proteins were inputted directly into the String database (https://string-db.org). Clusters were formed by selecting the MCL clustering option with an inflation parameter of 3 (default). Network edges were selected to indicate the strength of the data support. Finally, nodes disconnected from the network were hidden.

### Reporting summary

Further information on research design is available in the Nature Research Reporting Summary linked to this article.

## Supplementary information

Supplementary Information

Description of Additional Supplementary Files

Supplementary Data 1

Reporting Summary

## Data Availability

We generated PRO-seq libraries for MCF10A cells with and without Nutlin-3a. MCF10A PRO-seq data generated for this study are available in GEO with accession numbers GSE142419. In addition, a number of publicly available data sets were utilized and analyzed. These data sets are available in the Short Read Archive (SRA) or ENCODE repository with accession numbers presented in Supplemental Data 1. Additional supporting data for figures is available at the Open Science Framework^83^.

## Code availability

TFEA is available for download at https://github.com/Dowell-Lab/TFEA and comes with *muMerge* integrated. Alternatively, *muMerge* can be downloaded independently at https://github.com/Dowell-Lab/mumerge. Definition files for Singularity and Docker containers are available in the TFEA GitHub repository. Usage of these containers is recommended to simplify dependency management. Finally, all data analysis conducted in preparation of this manuscript is available in Juypter notebook format at https://github.com/Dowell-Lab/TFEA/tree/master/Jupyter_Notebooks.
