# Supplementary material for: Transcription factor enrichment analysis (TFEA) quantifies the activity of multiple transcription factors from a single experiment
Source: Commun Biol. 2021 Jun 2;4:661. doi: 10.1038/s42003-021-02153-7 (PMC8172830; doi:10.1038/s42003-021-02153-7)
Supplement: Supplementary file 1 — Supplementary Information [file 42003_2021_2153_MOESM1_ESM.pdf]

Supplementary Material

Transcription factor enrichment analysis (TFEA) quantifies the activity of multiple transcription factors from a single experiment

Jonathan D. Rubin[1], Jacob T. Stanley[2], Rutendo F. Sigauke[3], Cecilia B. Levandowski[1], Zachary L. Maas[2], Jessica Westfall[4], Dylan J. Taatjes[1], Robin D. Dowell[2,4,5,*]

[1] Department of Biochemistry, University of Colorado, Boulder CO 80309 USA

[2] BioFrontiers Institute, University of Colorado, Boulder CO 80309 USA

[3] Computational Bioscience Program, Anschutz Medical Campus, University of Colorado, Aurora, CO 80045 USA

[4] Department of Molecular, Cellular and Developmental Biology, University of Colorado, Boulder CO 80309 USA

[5] Department of Computer Science, University of Colorado, Boulder CO 80309 USA

∗ Corresponding author: robin.dowell@colorado.edu

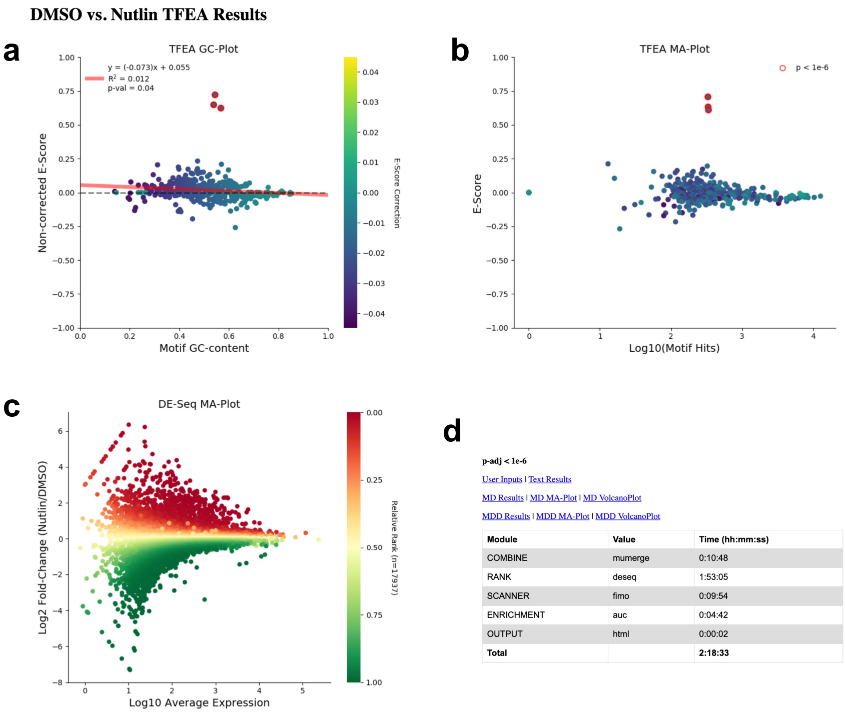

Module	Value	Time (hh:mm:ss)
COMBINE	mumerge	0:10:48
RANK	deseq	1:53:05
SCANNER	fimo	0:09:54
ENRICHMENT	auc	0:04:42
OUTPUT	html	0:00:02
**Total**		**2:18:33**

e

Positive E-Score

#TF	E-Score	Corrected E-Score	Events	GC	FPKM	P-adj	Corrected P-adj
P53_HUMAN.H11MO.0.A	0.722	0.707	331	0.545	9.07	1e-40	1e-39
P73_HUMAN.H11MO.0.A	0.648	0.633	333	0.539	2.19	1e-33	1e-31
P63_HUMAN.H11MO.0.A	0.624	0.61	337	0.568	0.0492	1e-31	1e-29
VDR_HUMAN.H11MO.0.A	0.209	0.195	324	0.57	2.42	1e-2	1e-1
FOXC1_HUMAN.H11MO.0.C	0.205	0.173	278	0.313	30.4	1e-2	1e-1
BACH2_HUMAN.H11MO.0.A	0.151	0.132	393	0.497	0.519	0.0752	0.207
FOSL1_HUMAN.H11MO.0.A	0.163	0.143	267	0.471	123	0.136	0.337
SMAD4_HUMAN.H11MO.0.B	0.15	0.135	313	0.557	8.43	0.186	0.352
JUND_HUMAN.H11MO.0.A	0.176	0.156	206	0.474	183	0.277	0.55
JUNB_HUMAN.H11MO.0.A	0.138	0.116	372	0.454	465	0.202	0.555
MAFK_HUMAN.H11MO.0.A	0.173	0.149	201	0.436	11.8	0.351	0.723
CUX1_HUMAN.H11MO.0.C	0.233	0.212	13	0.474	4.5	1	1
CEBPG_HUMAN.H11MO.0.B	0.188	0.162	122	0.397	13.7	0.669	1
MTF1_HUMAN.H11MO.0.C	0.167	0.155	57	0.599	5.45	1	1
ATF4_HUMAN.H11MO.0.A	0.173	0.147	115	0.399	517	1	1

⋮

Negative E-Score

#TF	E-Score	Corrected E-Score	Events	GC	FPKM	P-adj	Corrected P-adj
ZN121_HUMAN.H11MO.0.C	-0.0234	-0.0359	1.04e+04	0.583	27.4	1e-1	1e-5
FOXJ3_HUMAN.H11MO.0.A	-0.0391	-0.0799	3.27e+03	0.195	4.8	0.286	1e-4
ZN770_HUMAN.H11MO.0.C	-0.0205	-0.027	1.27e+04	0.664	36.5	1e-2	1e-4
THA_HUMAN.H11MO.0.C	-0.0492	-0.0653	4.26e+03	0.534	1.75	1e-2	1e-4
PAX5_HUMAN.H11MO.0.A	-0.0283	-0.0374	8.51e+03	0.629	0.179	1e-1	1e-3
PRDM6_HUMAN.H11MO.0.C	-0.0162	-0.0475	5.59e+03	0.325	0.22	1	1e-3
ZFP28_HUMAN.H11MO.0.C	-0.029	-0.0588	3.36e+03	0.346	0.0293	0.841	1e-2
IRF3_HUMAN.H11MO.0.B	-0.0158	-0.0401	6.3e+03	0.421	11.4	1	1e-2
VEZF1_HUMAN.H11MO.0.C	-0.0257	-0.0343	7.64e+03	0.636	26.9	0.115	1e-2
SP4_HUMAN.H11MO.0.A	-0.0397	-0.0457	4.74e+03	0.672	1.95	1e-1	1e-2
ZBT17_HUMAN.H11MO.0.A	-0.0331	-0.0423	5.46e+03	0.627	3.62	0.121	1e-1
ZSC22_HUMAN.H11MO.0.C	-0.0741	-0.0806	1.6e+03	0.664	7.11	0.0566	1e-1
KLF15_HUMAN.H11MO.0.A	-0.0298	-0.032	6.79e+03	0.724	1.31	0.0833	1e-1
WT1_HUMAN.H11MO.0.C	-0.0304	-0.0323	6.68e+03	0.728	0.0377	0.0799	1e-1
ZN281_HUMAN.H11MO.0.A	-0.0445	-0.0446	4.19e+03	0.752	24.3	1e-1	1e-1

⋮

Supplementary Figure 1: **An example of TFEA main HTML results page.** (a) Pre-GC correction showing the E-Score of each motif (y-axis) as a function of GC-content (x-axis). Red line: linear regression fit; dots colored by the amount to correct. (b) A scatter plot (colored as in a), similar to an MA-plot, showing the GC-corrected E-Scores (y-axis) vs the number of motif hits within regions (<1.5kb; x-axis) for each motif analyzed. (c) An MA-plot of the ROIs generated from DESeq2. (d) A table listing the inputs, text results, MD-Score (motif displacement score) and MDD-Score (differential motif displacement) results (as clickable links), as well as the time taken to complete each step of the TFEA process. (e) A list of motifs that exhibit positive (left) or negative (right) enrichment ordered by adjusted p-value. Significant motifs appear as red and have clickable links (blue) to individual results pages with more detailed information (see Supplementary Figure 2). List are truncated for readability. Data is HCT116 dataset, as used in Figure 3a[1].

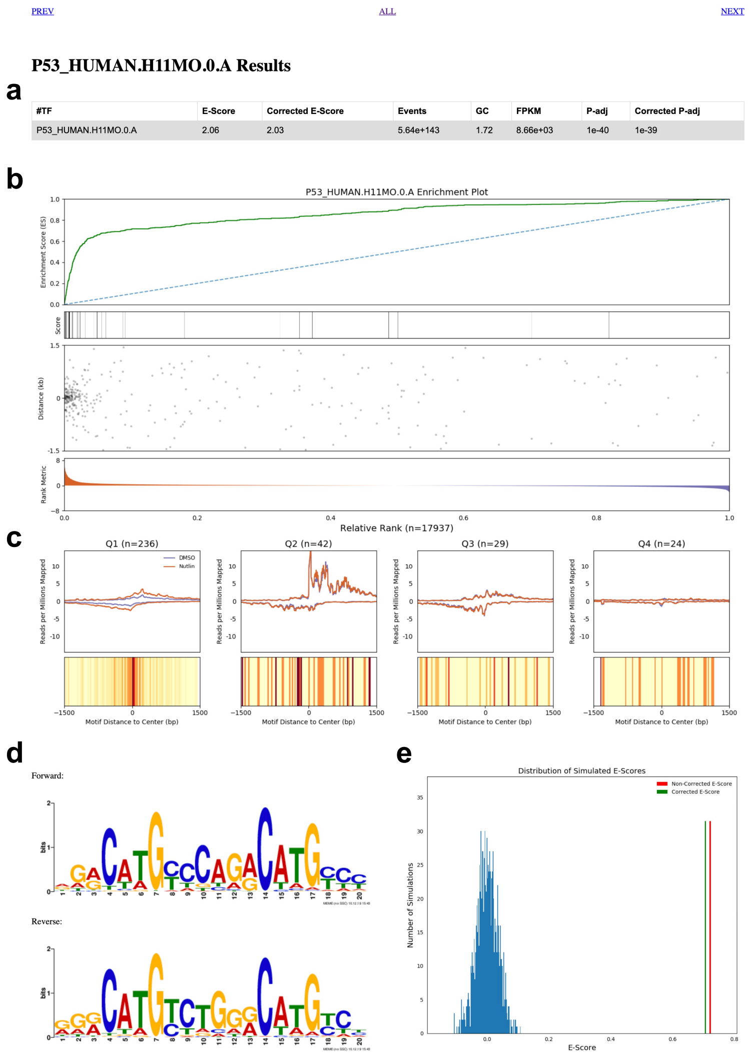

Supplementary Figure 2: **An example of a TFEA individual motif results page.** This page is reached by clicking on the corresponding motif in Supplementary Figure 1e. (a) Summary statistics for the motif of interest, in this case p53 from HOCOMOCO v11[8]. E-Score: Enrichment Score, Corrected E-Score: Enrichment score after GC correction using linear regression, Events: Number of motif hits within all ROIs, GC: GC content of motif, FPKM: Fragments per kilobase per million (with respect to the gene associated with the TF), P-adj: adjusted p-value of the E-Score, Corrected P-adj: adjusted p-value of corrected E-score. (b) Enrichment plots showing (from top to bottom) the running sum statistic (green line), the individual scores of each ROI (as a heatmap, darkness is greater score), scatter plot of motif hits within ROIs relative to the reference point (labeled 0), and the ranking of ROIs based on differential transcription (red: positive; blue: negative). (c) For each quartile, summarize motif containing ROI within the quartile via Top: Meta plot of read coverage over ROIs. Bottom: Motif displacement distribution (as heatmap: red is max; yellow is min) summarizing the motif positions relative to the reference point. (d) Logos of forward and reverse complement position specific scoring matrix of the motif analyzed. (e) Histogram of E-Scores from randomly shuffling the rank order of ROIs (blue) with true non-corrected E-score (red) and GC-corrected E-score (green).

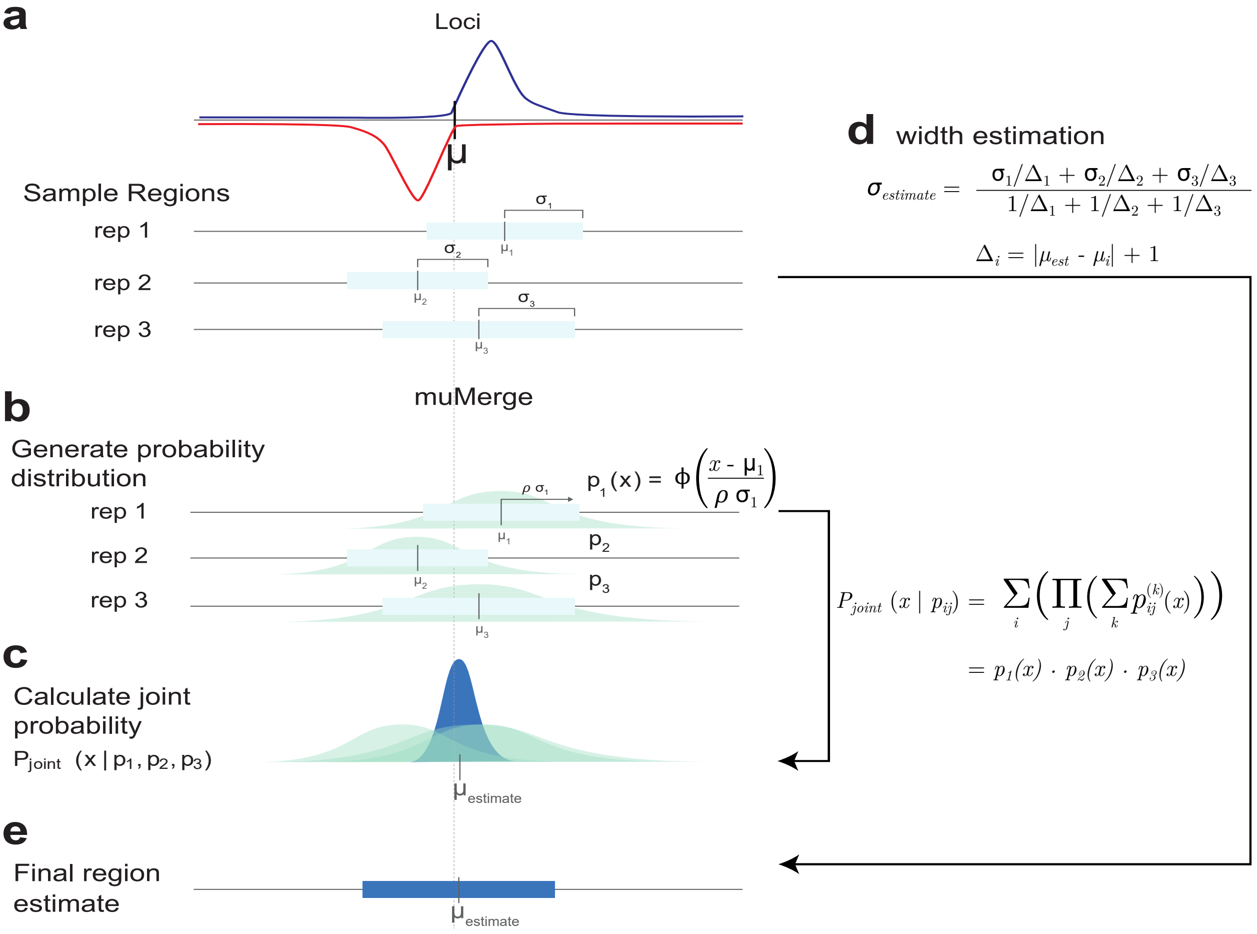

Supplementary Figure 3: **Diagrammatic description of the *muMerge* method.** (a) The goal of *muMerge* is to combine multiple sample regions (light blue boxes)—centers μ_i and half-widths σ_i—which originate from different replicates and/or conditions (rep 1,2,3), but are measurements of the same underlying loci μ, into a consensus set of regions of interest (ROIs). Red and blue lines represent a hypothetical bidirectional signal centered at μ. (b) *muMerge* assumes that each sample region is an estimate on the location of a genomic locus of interest and models this probability (p_i) as a normal distribution (ϕ, light green distributions) centered on the middle of each sample region μ_i, and standard deviation related to the region's half-width σ_i (scaled by ratio parameter ρ—default $= 1$). (c) Subsequently, a joint probability (p_{joint}, dark blue distribution—Eq. 2 in main text), is calculated from the sample distributions (k-index sum over within-sample peaks, j-index product over within-condition replicates, and i-index sum over conditions), and the estimate for the consensus position ($\mu_{estimate}$) is the local maxima of this joint distribution. (d) Next, to calculate the best estimate for the width of the ROI, a weighted average of the original sample region widths is calculated ($\sigma_{estimate}$)—Eq. 3 in main text. It is assumed that the sample regions closest to the consensus position are the most accurate representation of the underlying locus, so the weighted average of the widths is calculated such that more weight ($1/\Delta_i$) is given to the sample regions closer to $\mu_{estimate}$. (e) Thus, the final *muMerge* region estimate (dark blue box) is given by $(\mu_{est} - \sigma_{est}, \mu_{est} + \sigma_{est})$. The method is described in detail in Methods section "Defining ROIs with *muMerge*".

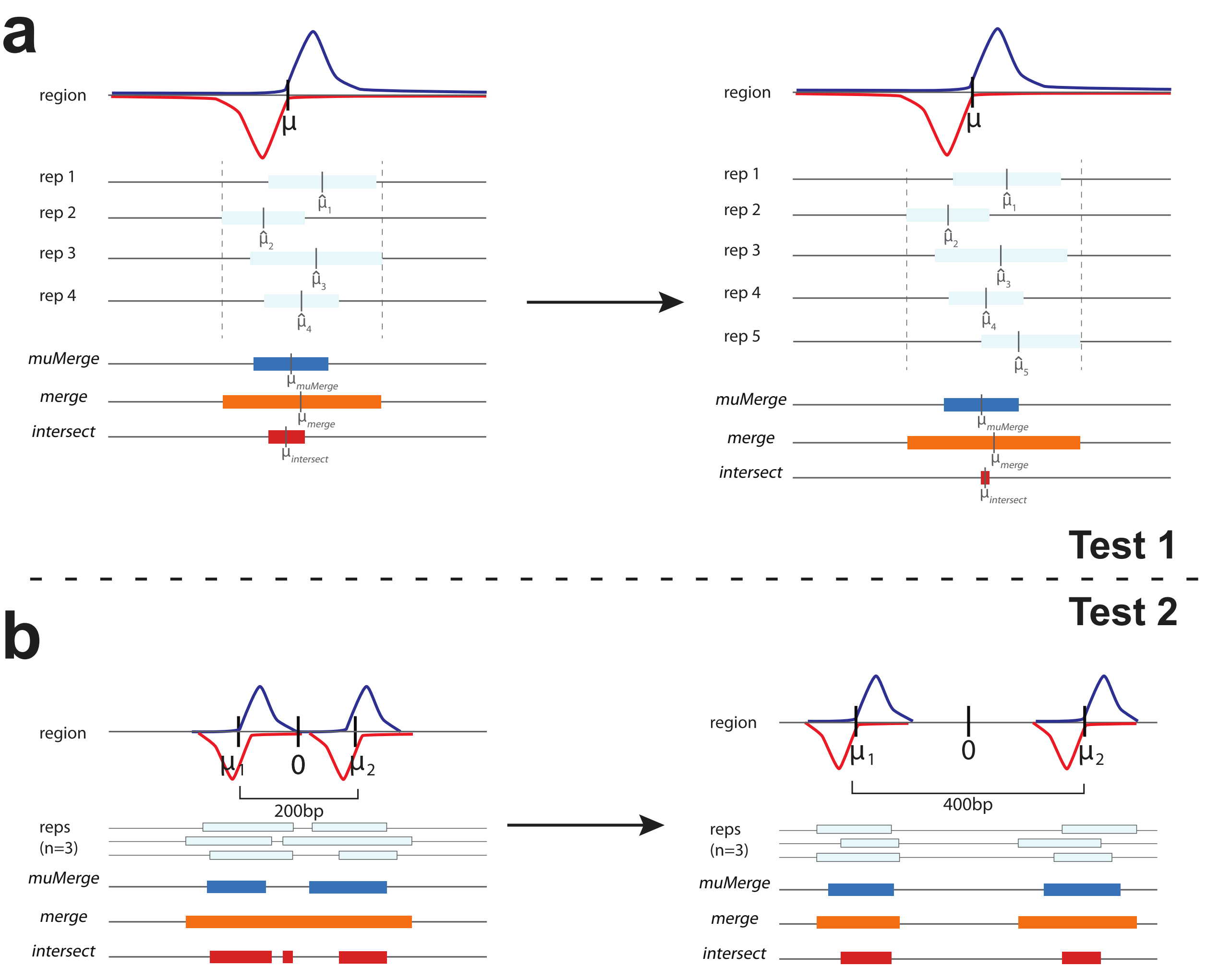

Supplementary Figure 4: **Tests to compare the performance of *muMerge* to that of *bedtools merge* and *bedtools intersect*.** The results of these two tests are shown in Figure 2. (a) The first test involves sampling regions from a single theoretical locus with increasing number of replicates (light blue boxes). *muMerge* (dark blue) retains correct length and μ position, while *bedtools merge* (orange) tends to increase ROI length and *bedtools intersect* (red) tends to decrease ROI length with increasing number of replicates. The quantitative results of this test are shown in Figure 2b of the main text. (b) The second test to determine performance involves sampling from two theoretical loci as a function of inter-locus spacing ($|\mu_2 - \mu_1|$) (light blue). For closely spaced loci, *muMerge* (dark blue) correctly separates the two loci whereas *bedtools merge* (orange) is more likely to generate a single ROI, and *bedtools intersect* (red) is more likely to generate multiple separate ROI (in this example, three). The quantitative results of this test are shown in Figure 2c of the main text. For both tests, the top cartoon depicts bidirectional signal on two strands (blue: positive strand; red: negative strand). Regions inferred from individual replicates in light blue. ROI ascertained by *muMerge* (dark blue), *bedtools merge* (orange) and *bedtools intersect* (red) shown for comparison.

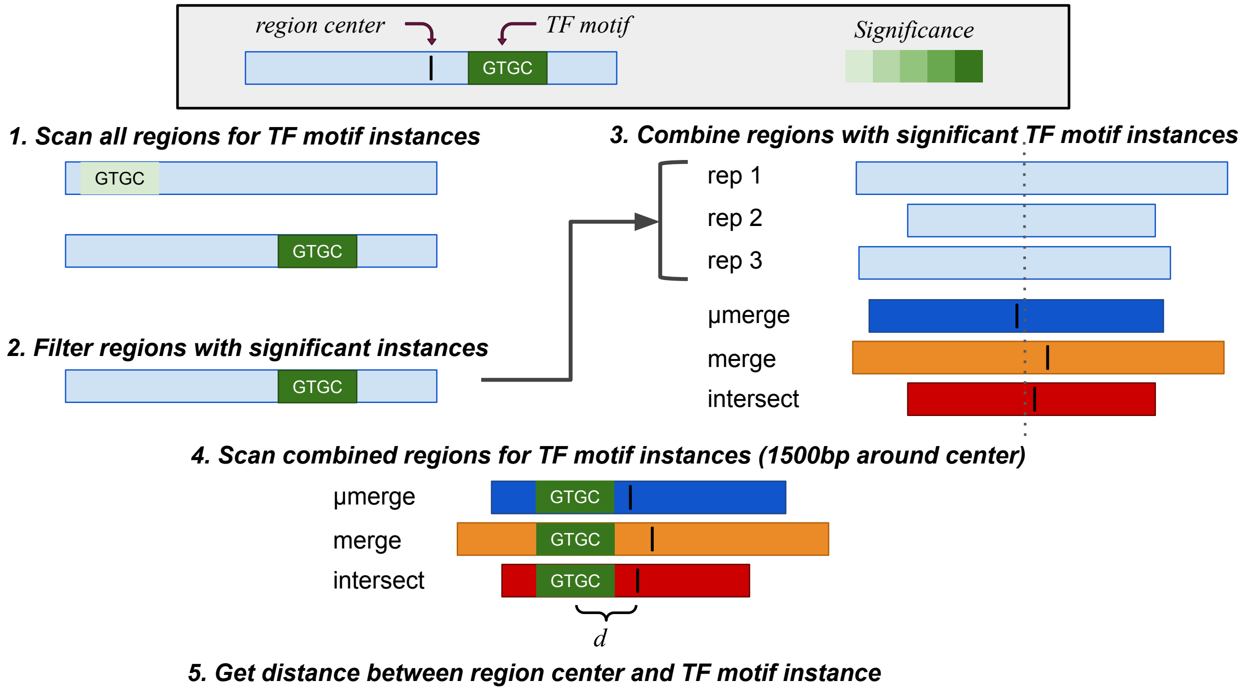

Supplementary Figure 5: **Schematic for method comparing *muMerge* (dark blue) with *bedtools merged* (orange) and *bedtools intersect* (red) on TF ChIP-seq data.** (1) Given ChIP-seq peak call regions (light blue boxes), the first step is to scan for TF motif instances (green) for all samples with FIMO[6]. (2) Peak regions with significant TF motif hits (dark green) (p-adj $<$ 0.001) are retained, and (3) significant replicate regions are combined with either *muMerge* (dark blue), *bedtools merge* (orange) and *bedtools intersect* (red)[10]. (4) Combined regions are expanded $\pm$1500 bp around the center (black vertical line) of the region and TF motif instances are determined. (5) Finally, the distance between the region center and the center of the best motif instance is calculated.

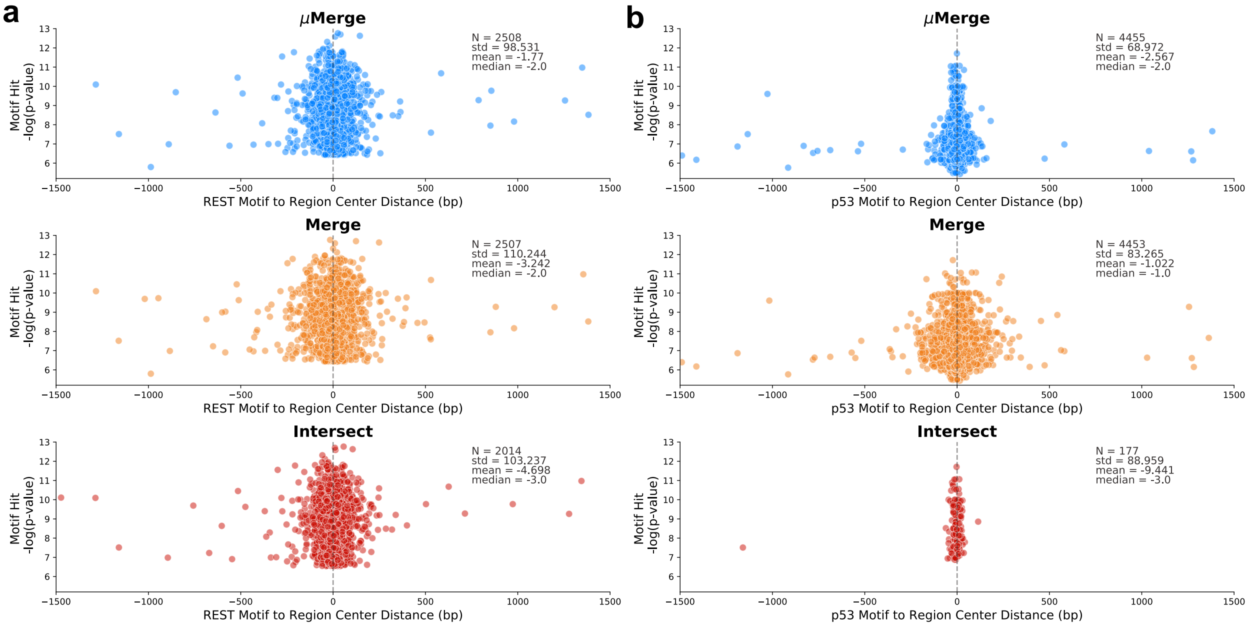

Supplementary Figure 6: **Results of comparison of *muMerge*, *bedtools merge*, *bedtools intersect* using ChIP-seq data for REST and p53.** Using the procedure of Supplementary Figure 5, scatter plots show the distance of the centers of ROIs (inferred, using the three methods, from identified ChIP peaks) to the corresponding TF binding motif. (a) Two REST[5] ChIP-seq replicates were combined using *muMerge* (dark blue), *bedtools merge* (orange) or *bedtools intersect* (red). The distance between the midpoint of the resulting region and the midpoint of the best motif instance is plotted (x-axis) relative to the motif score (y-axis). We note the mean is closest to zero for *muMerge* (dark blue) which also has the smallest standard deviation (std). This scenario has only two replicates which produces the smallest difference between the methods (consistent with Fig. 2b), since two samples is the least amount of replicate statistical power. However, *muMerge* still outperforms the other two methods—smallest standard deviation and mean closest to zero. (b) Similar comparison for p53[2] where cell types (HCT116, MCF7, and SJSA) are used as replicates (one sample per cell type) and combined across two conditions (DMSO and Nutlin-3a)—six samples in total. In this case, with multiple conditions, greater number of replicates, and “noisier” data (i.e. multiple cell lines), *muMerge* (dark blue) significantly outperforms the other two methods—*bedtools merge* (orange) produces large deviation from the motif while *intersect* (red) is only able to infer non-zero ROI for ChIP peaks in both conditions, which happen to correlate with highly significant motif instances (p-value $< 10^7$), missing $> 97\%$ of the ROI identified by the others ($N = 177$ vs. $N \sim 4450$). Conversely, *muMerge* infers ROIs for a broad range of motif instance significance, and demonstrates the lowest deviation from the motif location (standard deviation ~ 69 bases). Example regions for the p53 comparison are shown in Supplementary Figure 7. See Supplementary Data 1 for complete list of accession numbers for data utilized.

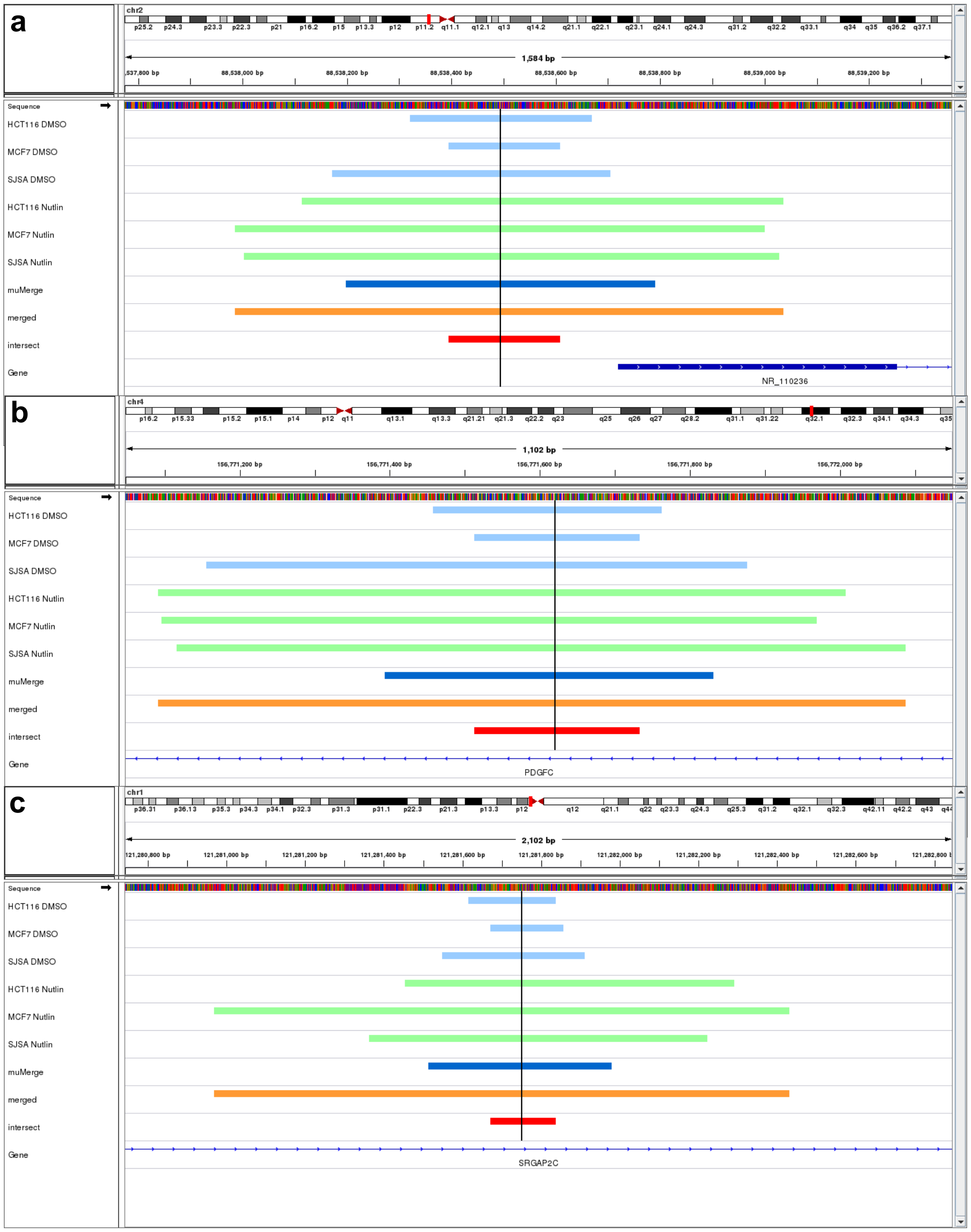

Supplementary Figure 7: **Examples of *muMerge* (dark blue) performance compared with *bedtools merge* (orange) and *bedtools intersect* (red) for three ChIP peaks (displayed in IGV).** Three regions (a-c) from the p53 comparison of Supplementary Figure 6 are shown for the three cell types (treated as replicates) and two conditions (DMSO: light blue and Nutlin-3a: green). In all three cases, the motif location (vertical black line) is accurately inferred by all three methods. Consistent with the results in Figure 2b/c (and depicted in Supplementary Figure 4), *merge* represents the upper limit on the ROI size and *intersect* represents the lower limit. Conversely, *muMerge* strikes a balance between these two extremes.

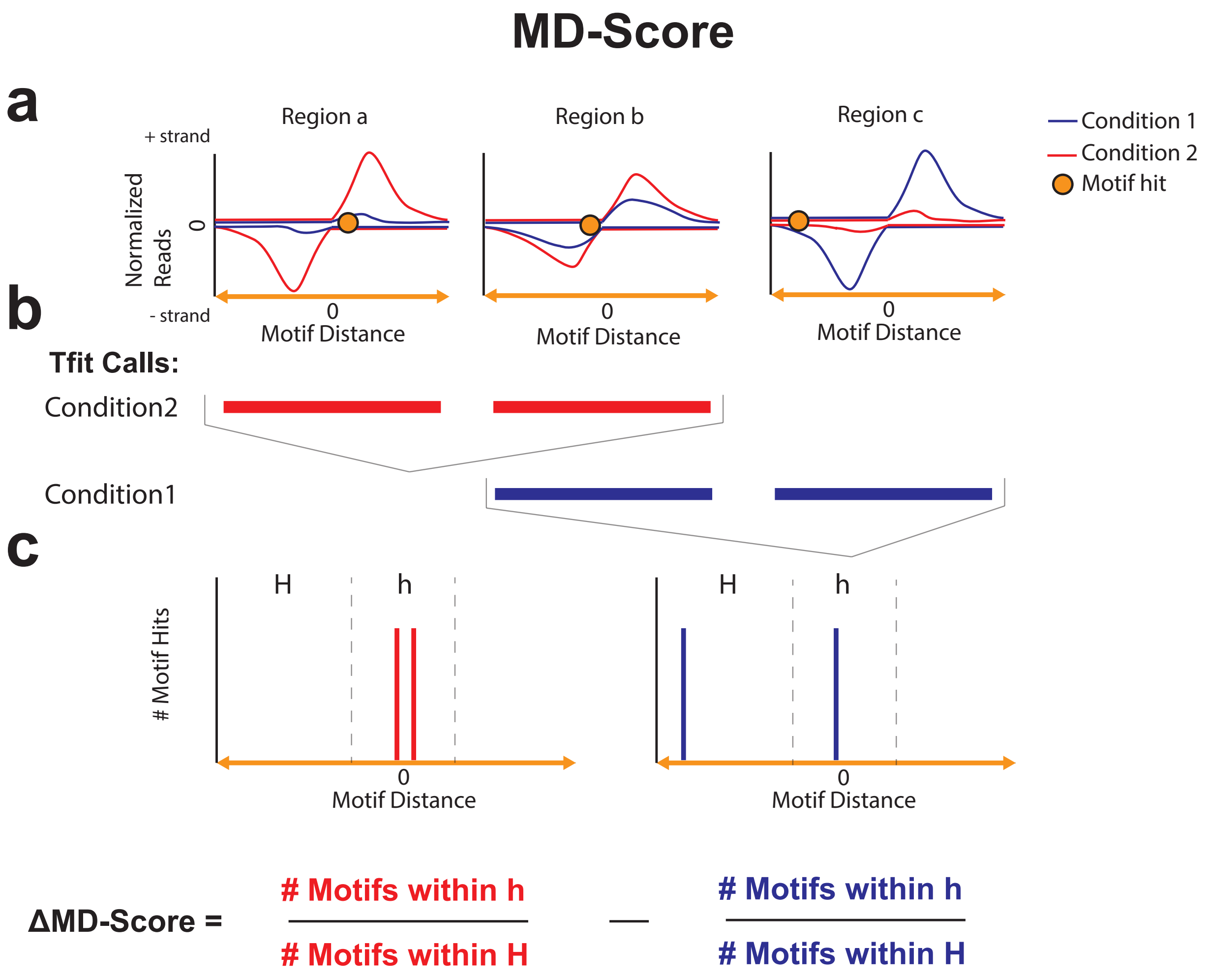

Supplementary Figure 8: **Schematic description of MD-Score method.** (a) Cartoon depicting typical histograms of nascent transcription data (condition 1: blue, condition 2: red) for three example regions (regions a, b and c). Orange dot represents a TF motif instance. (b) Regions of RNA polymerase initiation identified in each dataset (red, blue boxes), for example as called by Tfit[4]. These regions are the inputs to the MD-score (motif displacement score) approach[3]. (c) Motif displacement distribution histograms plot position of motif (vertical bars) relative to reference point (labeled 0) for both conditions (red and blue). The MD-Score is the fraction of motif instances within the inner window (h=150 bp) divided by the total motif hits in the larger window (H=1500 bp; note H encompasses h). MD-Scores are calculated independently in each of the two conditions to obtain the difference.

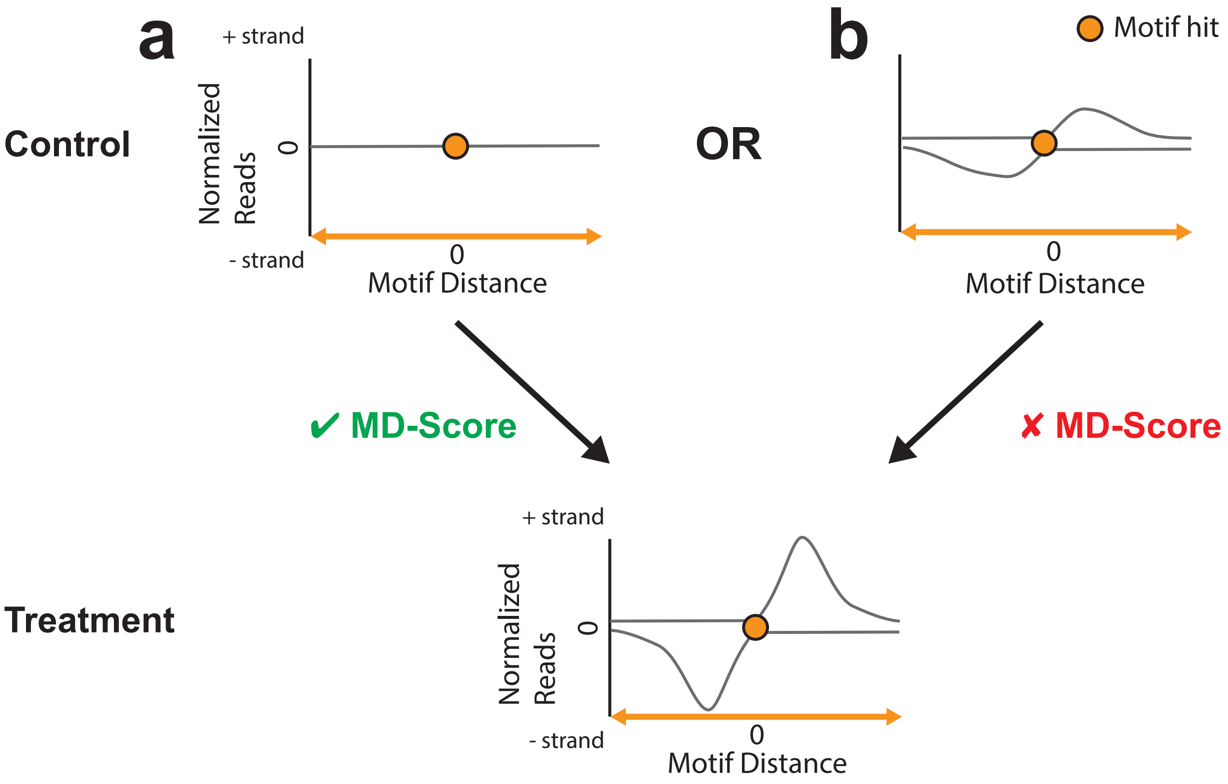

Supplementary Figure 9: **The MD-Score approach only detects gain or loss of transcribed regions.** A given locus in the treatment can arise from either (a) a region of no signal in the control; or (b) increase in signal at a pre-existing region within the control sample. Importantly, the first case increases the Δ MD-Score whereas the second does not.

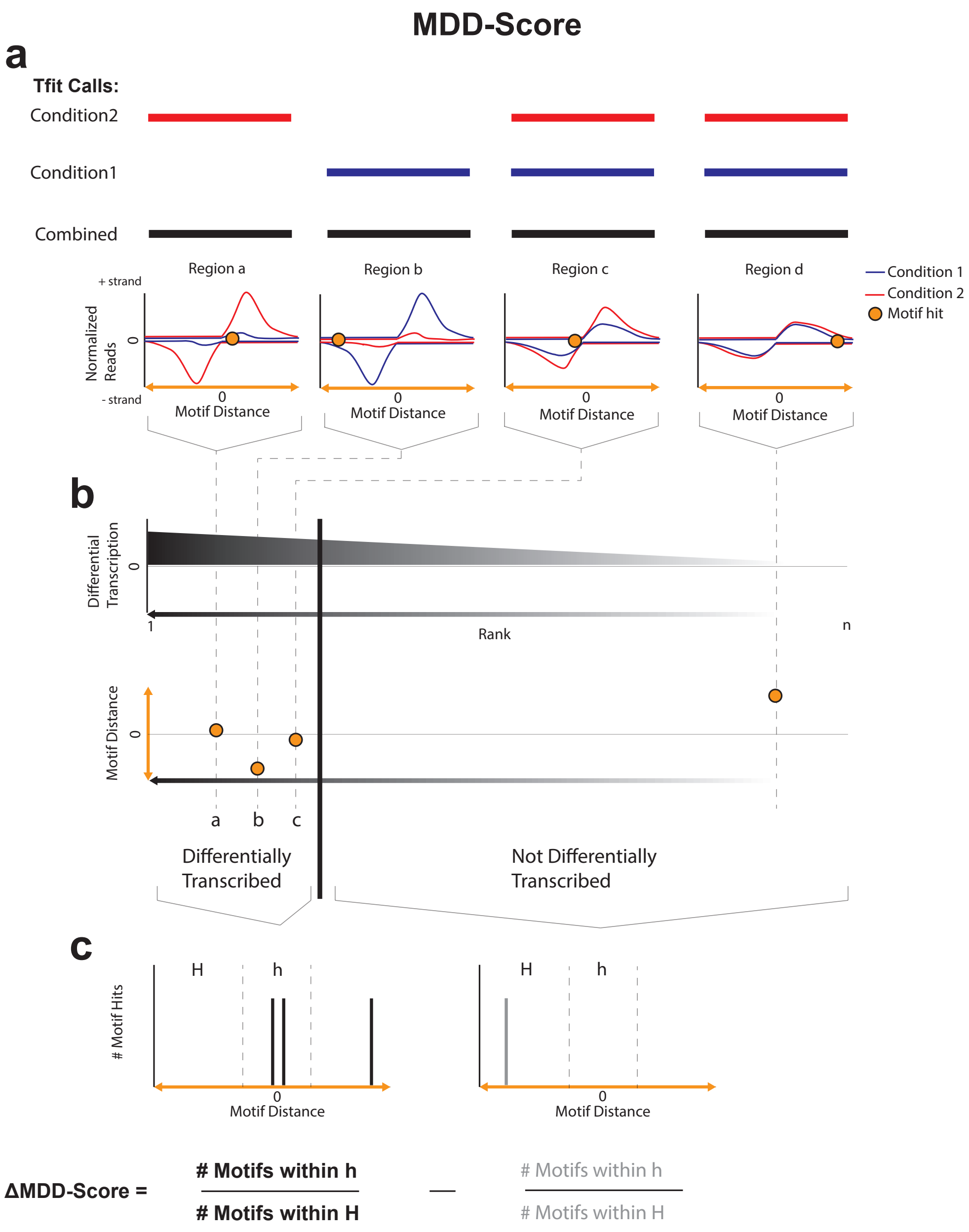

Supplementary Figure 10: **Schematic depicting the MDD-Score method.** The differential MD-Score method (referred to as MDD-Score)[11, 7] begins with (a) a collection of regions called in one or more conditions (red and blue boxes). Combined regions (black) are then (b) ranked by DESeq or DESeq2 p-value (depending on replicate number) and a cutoff segregates the differentially transcribed subset. (c) The MDD-Score is then the difference of MD-Score between the differentially transcribed set (black histogram) and the not differentially transcribed (grey histogram).

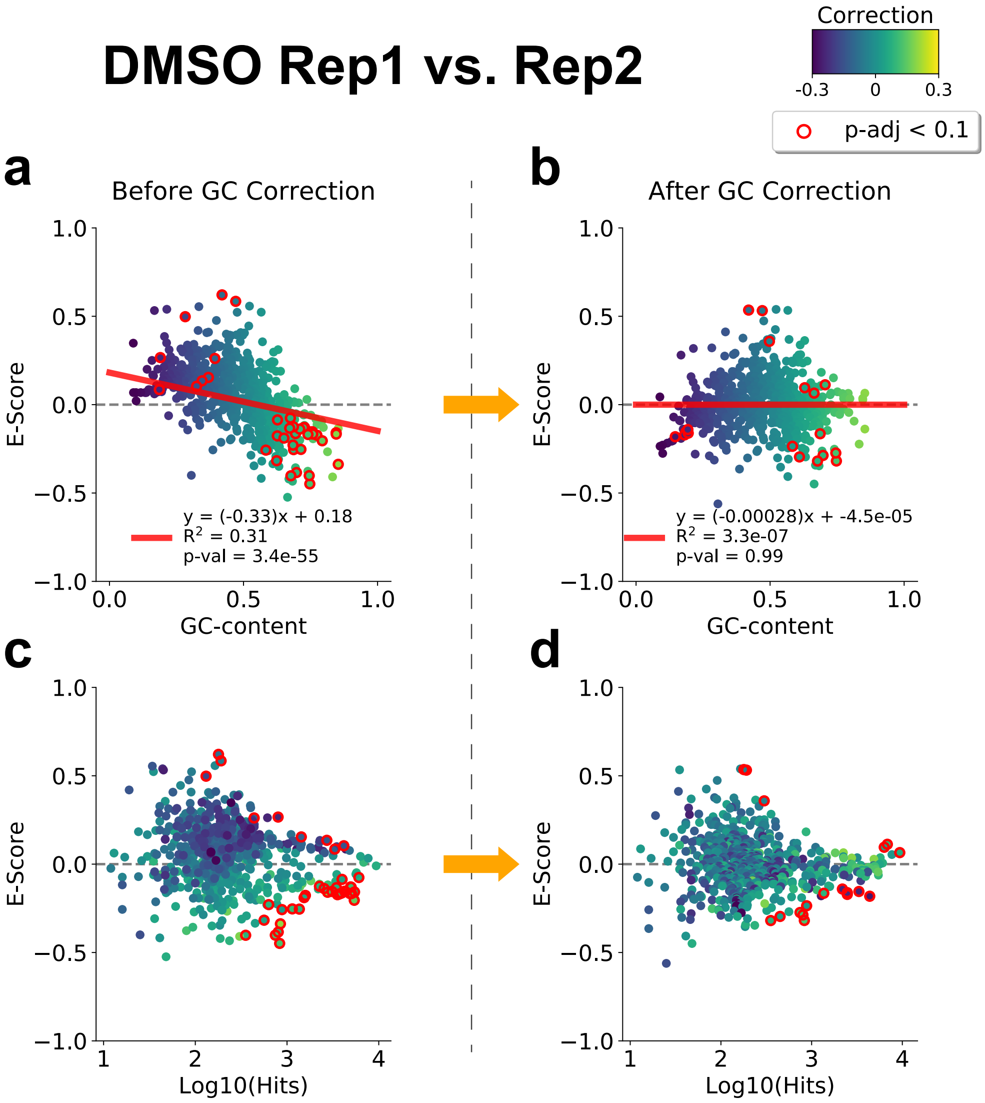

Supplementary Figure 11: **Enrichment scores (E-Scores) are adjusted based on the GC content bias using linear regression.** We observed that motif E-Scores often correlated with their GC-content. (a) Scatter plot of E-Score (y-axis) vs. GC-content (x-axis) of motifs, comparing replicate 1 vs. replicate 2 (DMSO condition) before GC-correction (red line: linear regression fit). (b) Scatter plot of E-Score (y-axis) vs. GC-content (x-axis) of motifs after GC correction (red line: linear regression fit). (c) MA plot of E-Score (y-axis) vs. Log10 number of motif hits within regions of interest (x-axis) before GC correction. (d) MA plot of E-Score (y-axis) vs. Log10 number of motif hits within regions of interest (x-axis) after GC-correction. These MA plots show that the underlying distribution of E-Scores relative to number of motif hits does not significantly change after GC-correction. All panels are data in HCT116 DMSO condition (SRR1105736, SRR1105737 [1]), dots are colored by the amount to be corrected due to GC-bias, red outline dots are p-adj $<$ 0.1.

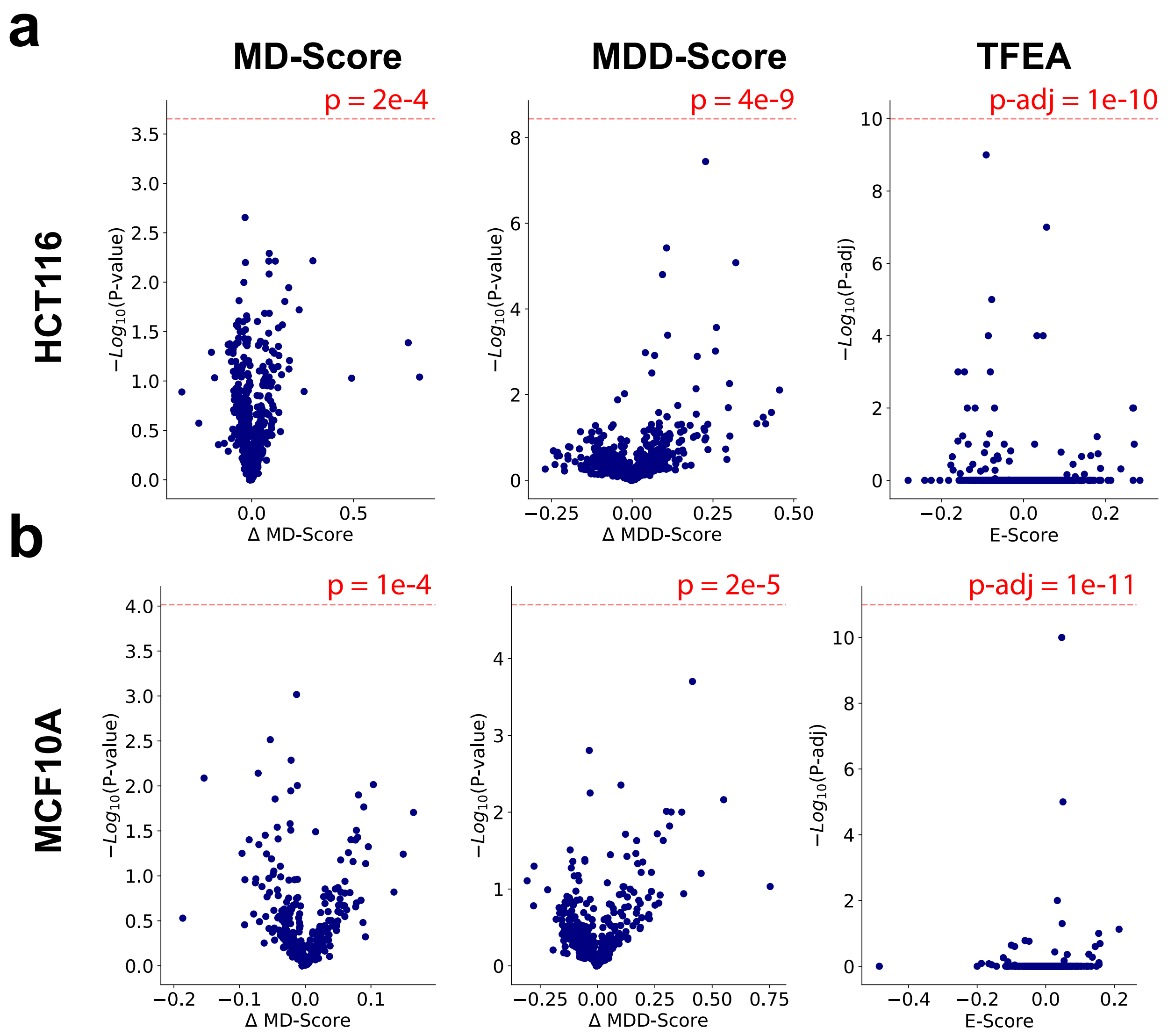

Supplementary Figure 12: **Choosing thresholds for MD-Score, MDD-Score, and TFEA.** To choose a threshold cutoff for each of the three methods, DMSO replicates were compared and the threshold at which no false positives are obtained was determined. To be conservative, an additional order of magnitude is added for stringency. We performed this for each method in both (a) HCT116 and (b) MCF10A cells.

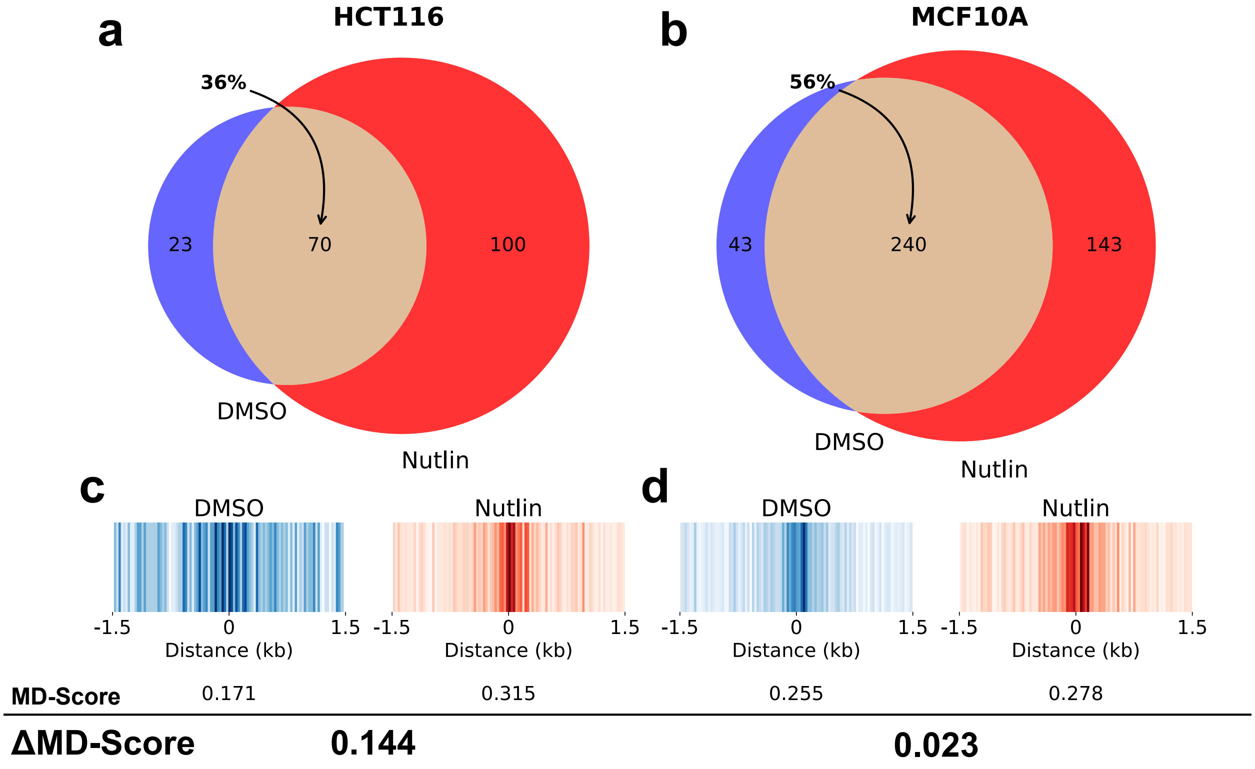

Supplementary Figure 13: **The MD-score approach fails to capture p53 after Nutlin-3a treatment in MCF10A cells.** The response to Nutlin-3a visualized as Venn diagrams of (a) HCT116 and (b) MCF10a cells show a distinct p53 response, with a larger proportion (in MCF10A cells) of existing sites of RNA polymerase initiation (DMSO, blue) that respond to Nutlin-3a (red; overlap shown in tan). In both cases, only regions with p53 motif within 150 bps of the point of interest (midpoint of ROI) are shown. Motif displacement distributions of TP53 motif within 1.5 kb of ROI midpoints for (c) HCT116 or (d) MCF10A cells in DMSO (blue) and Nutlin-3a (red) conditions shows a higher co-localization of p53 in DMSO treated MCF10A cells. Bottom: MD-Score quantification for each condition followed by the observed ΔMD-Score for the Nutlin-3a response in each cell type.

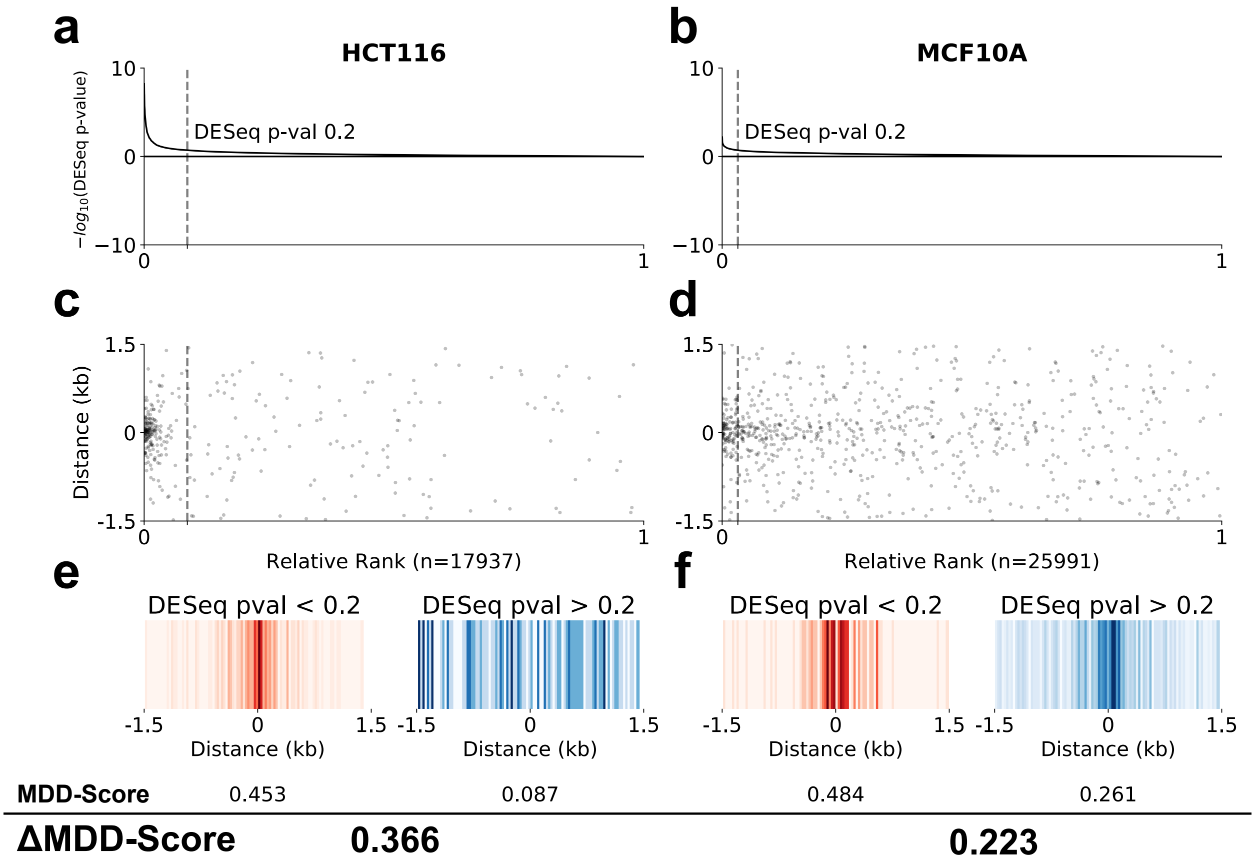

Supplementary Figure 14: **The MDD-Score method detects p53 following Nutlin treatment in both cell types.** The MDD-Score approach detects p53 response in both (a) HCT116 and (b) MCF10a cells. By default, a loose DESeq2 p-value of 0.2 is chosen to identify the set of differentially transcribed ROI (similar to [11]). Scatterplots show instances of TP53 motif across ranked ROI for (c) HCT116 and (d) MCF10A cells. The presence of constitutive TP63 activity leads MCF10a cells to have a higher background signal around TP53 motifs. Motif displacement distribution heatmaps for (e) HCT116 and (f) MCF10A cells, further emphasize the increased background presence of the TP53 motif in MCF10A cells. Red is control (DMSO), blue is Nutlin-3a treated. HCT116 data from SRR1105736, SRR1105737, SRR1105738, SRR1105739.

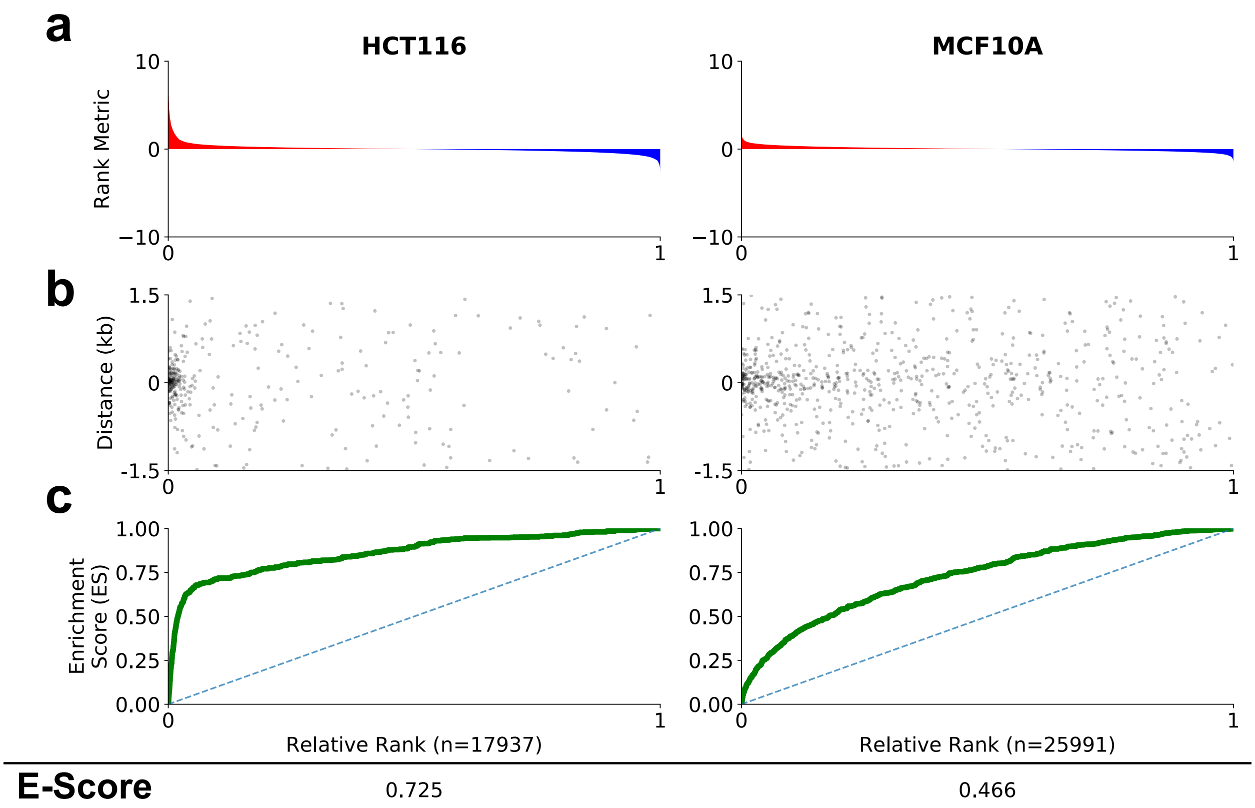

Supplementary Figure 15: **TFEA detects p53 in both HCT116 cells and MCF10A cells without the use of fixed thresholds.** (a) ROI are ranked by differential transcription. Red: increased transcription, blue: decreased. (b) Instances of the TP53 motif are detected within ranked ROIs. (c) TFEA measures motif enrichment as the E-Score, calculated as 2* AUC (ie. area under the curve) between the running sum of ROI scores (green line) and the uniform distribution (dashed blue line). HCT116 data from SRR1105736, SRR1105737, SRR1105738, SRR1105739.

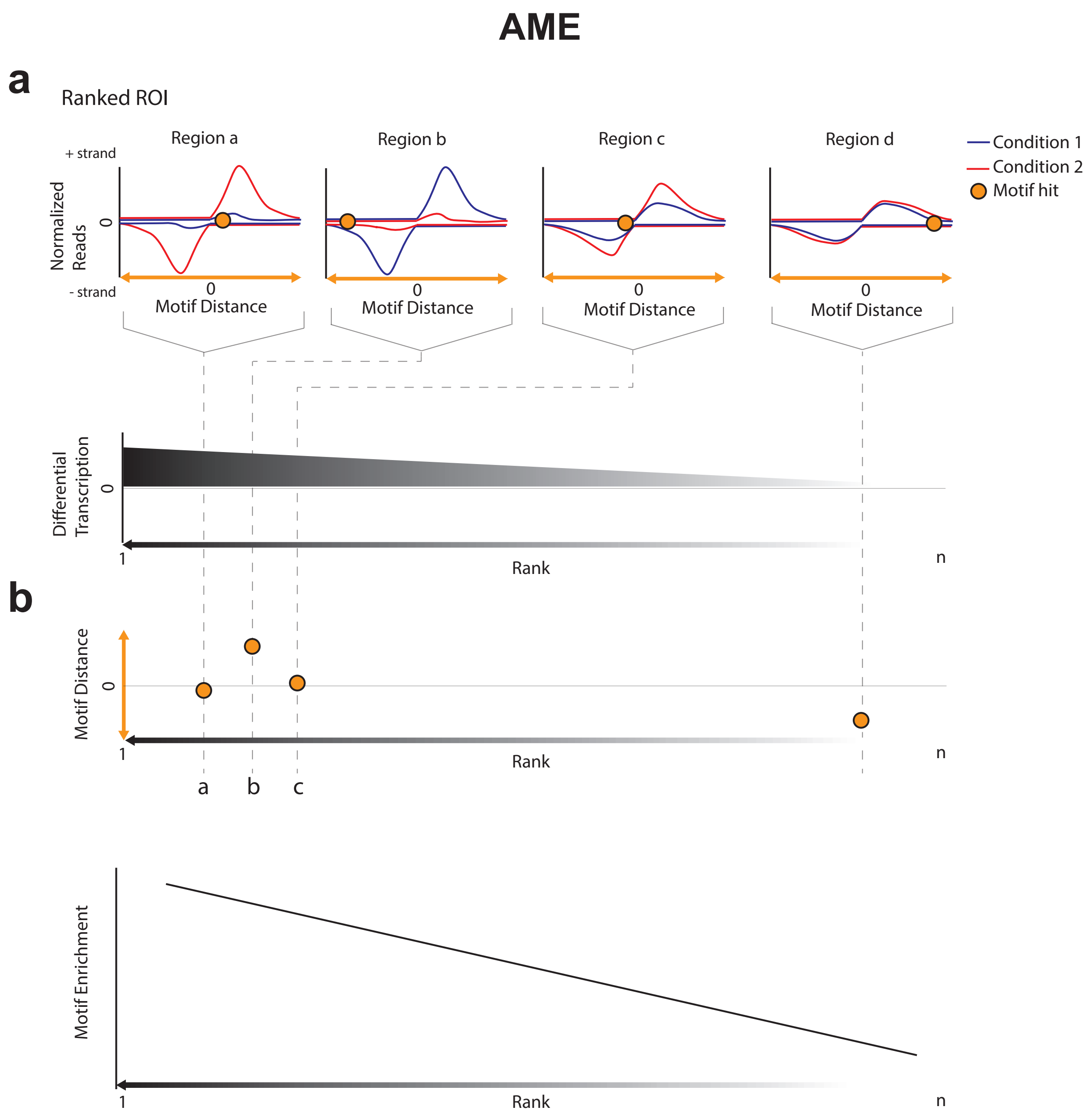

Supplementary Figure 16: **Schematic depicting the AME method.** Analysis of Motif Enrichment (AME) is part of the MEME suite and requires (a) a ranked list of regions of interest (ROIs, labeled a-d) as input. AME then performs (b) linear regression on the motifs as a function of rank, ignoring the distance to motif (orange circles) information.

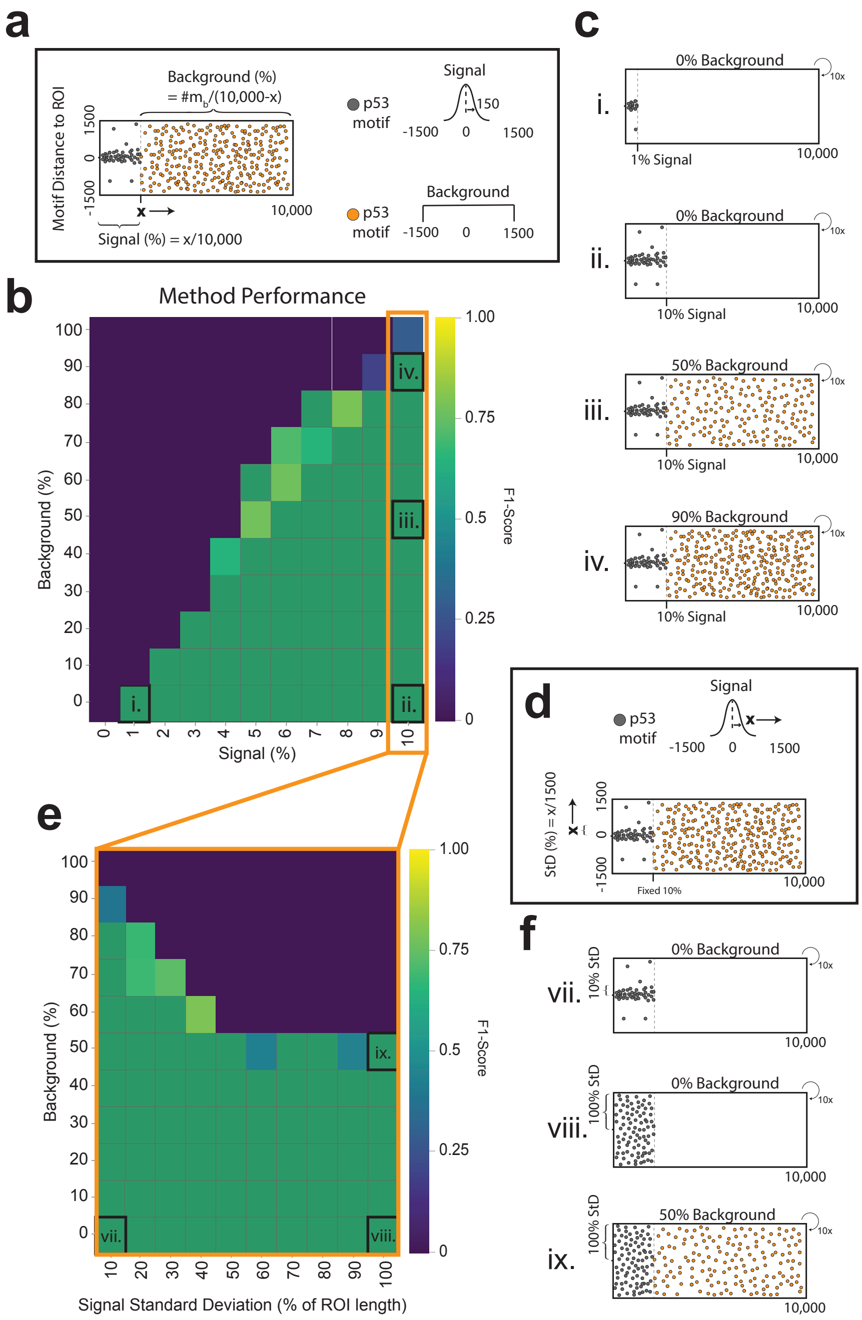

Supplementary Figure 17: **Diagram depicting the benchmark strategy utilized in Figure 4.** (a) A description of key concepts of motif embedding strategy for both signal (grey) and background (orange). (b) F1-Score (as heatmap) for varying fraction of ROI with signal (x-axis) and background (y-axis). Representative tests cases are labeled (i-iv) and their (c) respective embedding strategies are shown. (d) A description of additional criteria utilized for altering the variability of signal embedding. (e) For 10% signal, we additionally alter the signal standard deviation (x-axis) vs background (y-axis). Representative cases (vii-ix) are labeled and their (f) respective embedding strategies are shown.

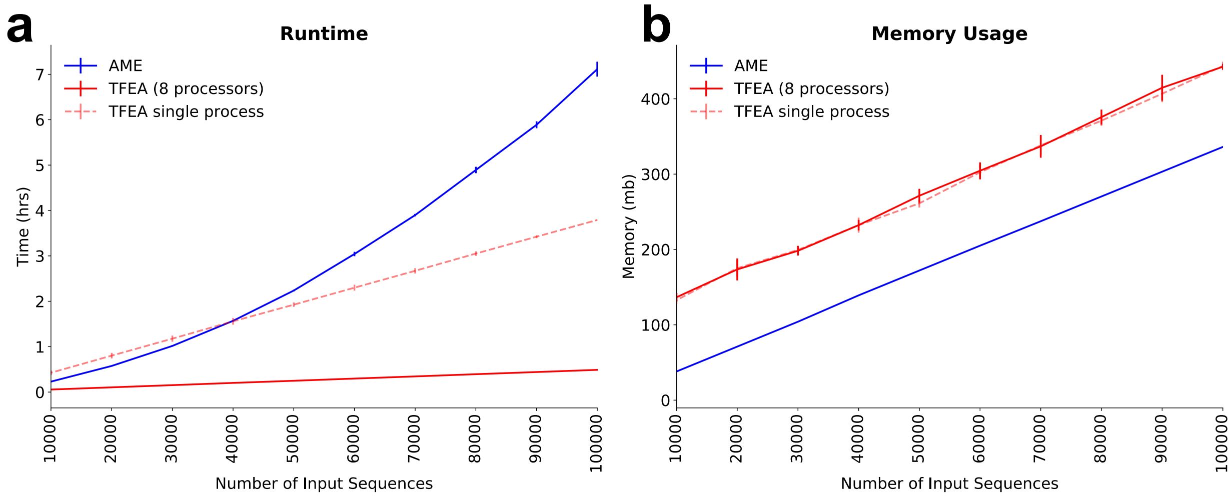

Supplementary Figure 18: **TFEA is fast and memory efficient.** (a) Runtime statistics for AME (solid blue; parallel processing not supported) and TFEA (8 processors: solid red; 1 processor: dashed red) with varying numbers of input ROI (bars = standard deviation of 10 runs). (b) Memory usage statistics comparing AME to TFEA.

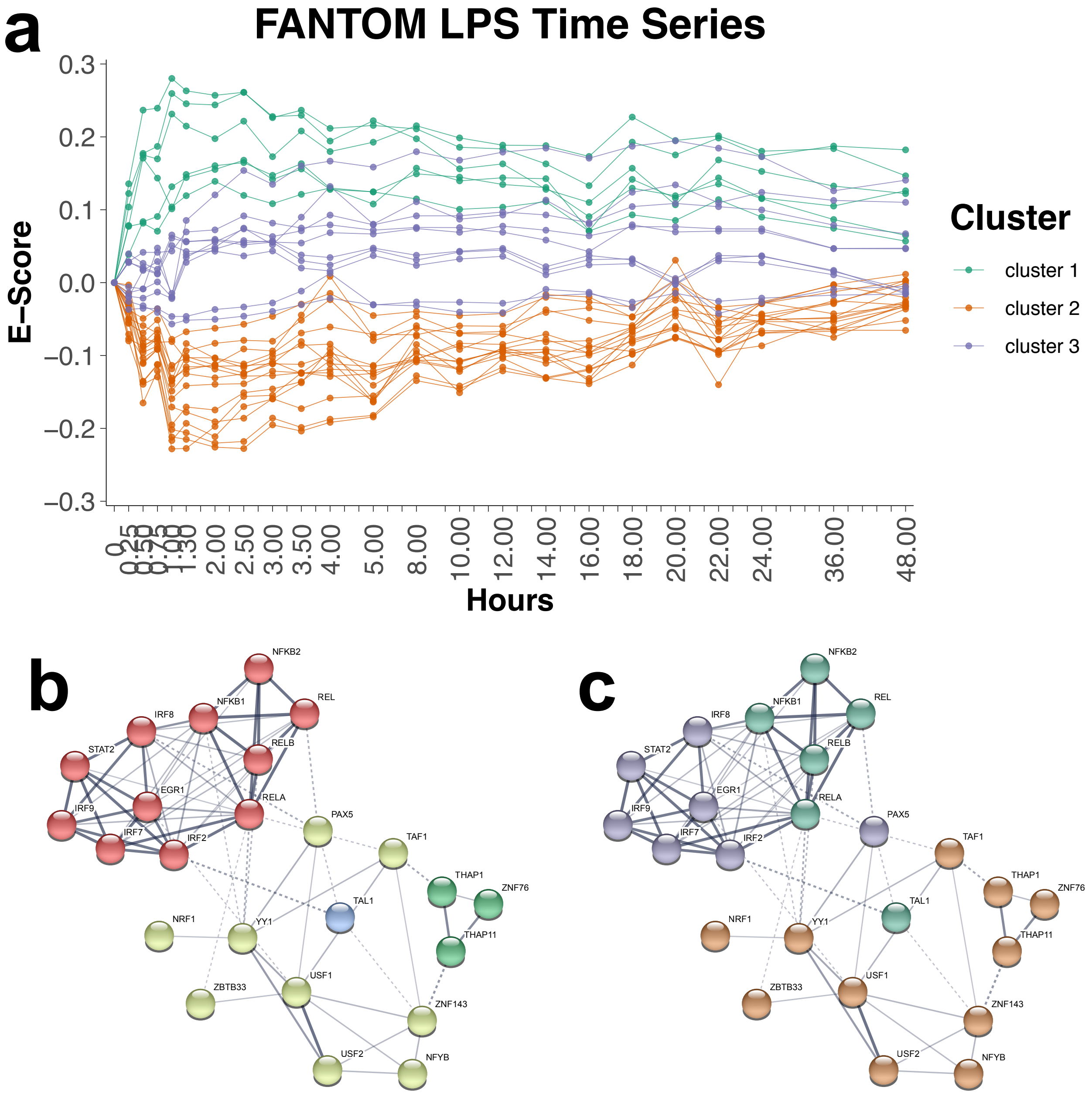

Supplementary Figure 19: **Clustering LPS induced TFs based on dynamics over time.** We applied k-means clustering to the subset of TFs that were significant (by TFEA) in at least 15 time points (~2/3 of all timepoints; n=32 TFs). (a) Time series traces of significant TFs colored by resulting cluster. The three main clusters correspond to the immediate increased response (cluster 1, green), the immediate decreased response (cluster 2, orange) and the later responding TFs (cluster 3, purple). (b) Alternatively the TFs can be analyzed using the String database using the Markov cluster algorithm. (c) Superposition of the coloring scheme in (a) onto the network cluster of (b).

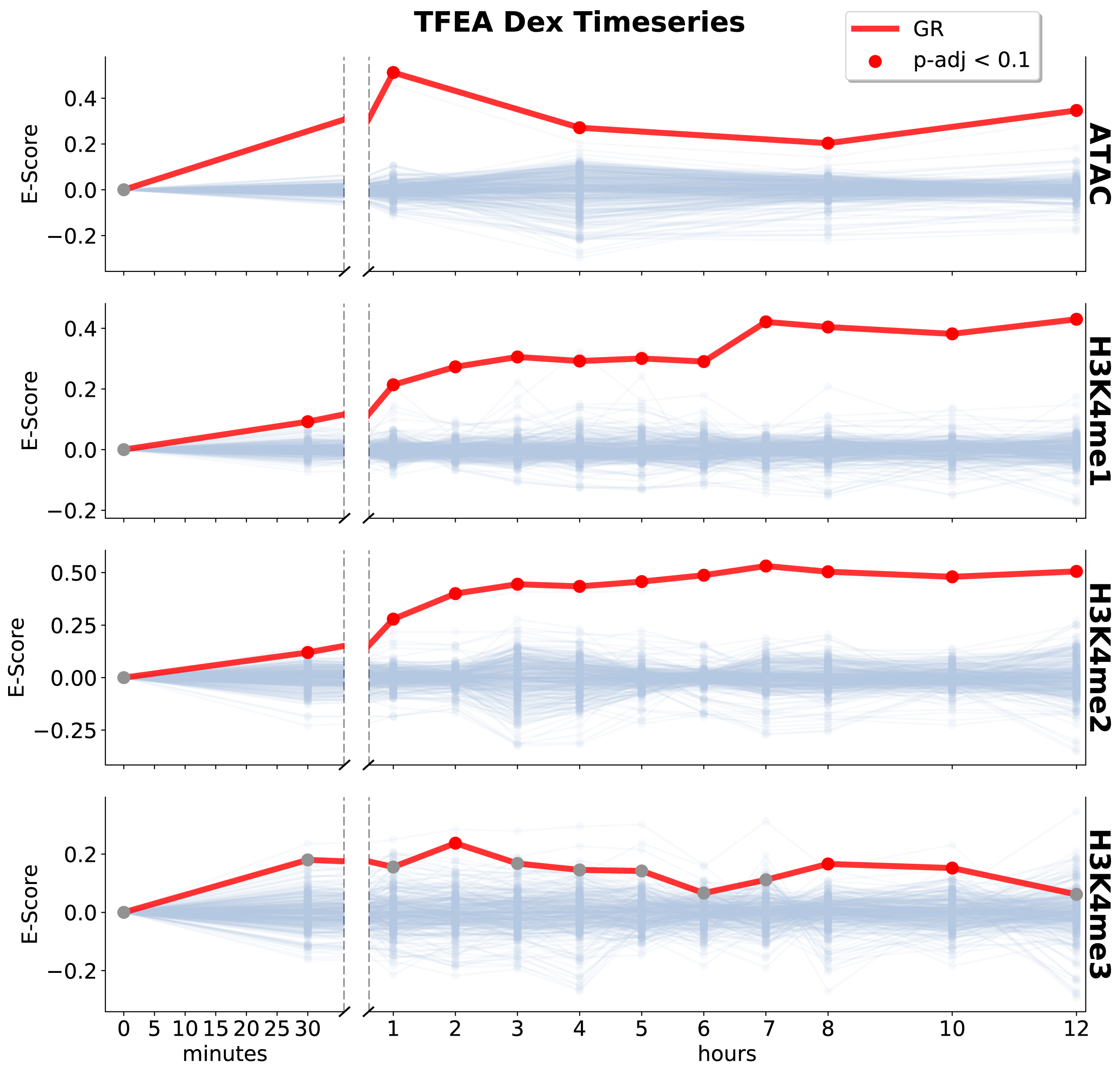

Supplementary Figure 20: **TFEA recovers the glucocorticoid receptor (GR) following treatment with Dexamethasone.** TFEA is able to recover GR (red line) in many distinct data sets including ATAC, H3K4me1, and H3K4me2. Interestingly, TFEA only detects moderate enrichment of GR in H3K4me3, in agreement which previous results indicating that GR primarily binds to enhancers (which do not have the H3K4me3 mark)[9]. Grey lines are trajectories of other TFs.

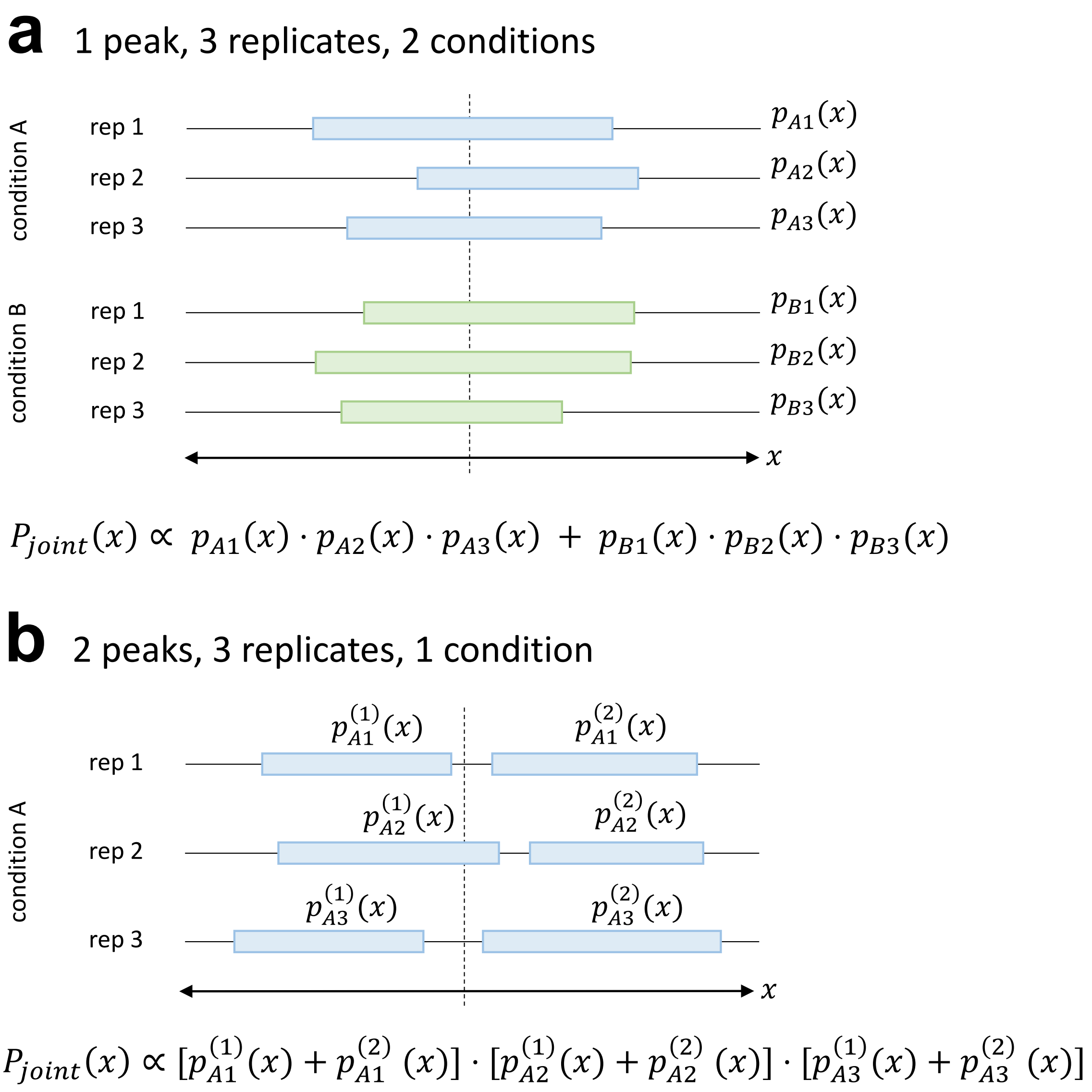

Supplementary Figure 21: **Examples of $\mathcal{P}_{joint}(x \,|\, p_{ij})$ calculations—Eq. 2 in the Methods**. Here we have two hypothetical examples of overlapping sample regions (rep 1,2,3) from two different experiments. The corresponding calculation of $\mathcal{P}_{joint}$ (from individual sample probability distributions $p_{ij}^{k}(x)$) is shown for both examples, as a function of genomic coordinate x. (a) A genomic location for an example experiment consisting of two conditions (condition A: blue, condition B: green), each of which has three replicates (rep). Each $p_{ij}^{(k)}(x)$ is the normal distribution representing the corresponding sample region—Eq. 1 in the Methods. (b) A genomic location for an example experiment consisting of a single condition, which has three replicates. This region contains two distinct (but closely spaced) loci in each replicate. NOTE: due to the positioning of individual sample regions, *bedtools merge* would produce a single, large ROI and *bedtools intersect* would produce three separate ROIs with the middle one being very narrow. Conversely, *muMerge* would produce two distinct ROIs—the ideal outcome for this particular example.

Data Utilized

See Supplementary Data 1.

Supplementary References

[1] M. A. Allen, H. Mellert, V. Dengler, Z. Andryzik, A. Guarnieri, J. A. Freeman, X. Luo, W. L. Kraus, R. D. Dowell, and J. M. Espinosa. Global analysis of p53-regulated transcription identifies its direct targets and unexpected regulatory mechanisms. *eLife*, 3:e02200, 2014. doi: 10.7554/eLife.02200.

[2] Z. Andrysik, M. D. Galbraith, A. L. Guarnieri, S. Zaccara, K. D. Sullivan, A. Pandey, M. MacBeth, A. Inga, and J. M. Espinosa. Identification of a core TP53 transcriptional program with highly distributed tumor suppressive activity. *Genome research*, 27(10):1645–1657, 2017.

[3] J. G. Azofeifa, M. A. Allen, J. R. Hendrix, T. Read, J. D. Rubin, and R. D. Dowell. Enhancer RNA profiling predicts transcription factor activity. *Genome Research*, 28(3):334–344, Feb 2018.

[4] J. G. Azofeifa and R. D. Dowell. A generative model for the behavior of RNA polymerase. *Bioinformatics*, 33(2):227–234, 09 2016.

[5] C. A. Davis, B. C. Hitz, C. A. Sloan, E. T. Chan, J. M. Davidson, I. Gabdank, J. A. Hilton, K. Jain, U. K. Baymuradov, A. K. Narayanan, K. C. Onate, K. Graham, S. R. Miyasato, T. R. Dreszer, J. S. Strattan, O. Jolanki, F. Y. Tanaka, and J. M. Cherry. The Encyclopedia of DNA elements (ENCODE): data portal update. *Nucleic Acids Research*, 46(D1):D794–D801, 2018.

[6] C. E. Grant, T. L. Bailey, and W. S. Noble. FIMO: scanning for occurrences of a given motif. *Bioinformatics*, 27(7):1017–1018, Apr. 2011.

[7] M. A. Gruca, M. A. Gohde, and R. D. Dowell. Annotation agnostic approaches to nascent transcription analysis: Fast read stitcher and transcription fit. *Methods in Molecular Biology*, to appear, 2020.

[8] I. V. Kulakovskiy, I. E. Vorontsov, I. S. Yevshin, R. N. Sharipov, A. D. Fedorova, E. I. Rumynskiy, Y. A. Medvedeva, A. Magana-Mora, V. B.

Bajic, D. A. Papatsenko, F. A. Kolpakov, and V. J. Makeev. HOCOMOCO: towards a complete collection of transcription factor binding models for human and mouse via large-scale ChIP-Seq analysis. *Nucleic Acids Research*, 46(D1):D252–D259, Jan. 2018.

[9] I. C. McDowell, A. Barrera, A. M. D'Ippolito, C. M. Vockley, L. K. Hong, S. M. Leichter, L. C. Bartelt, W. H. Majoros, L. Song, A. Safi, D. D. Koçak, C. A. Gersbach, A. J. Hartemink, G. E. Crawford, B. E. Engelhardt, and T. E. Reddy. Glucocorticoid receptor recruits to enhancers and drives activation by motif-directed binding. *Genome Research*, 28:1272–1284, Aug. 2018.

[10] A. R. Quinlan and I. M. Hall. BEDTools: a flexible suite of utilities for comparing genomic features. *Bioinformatics*, 26(6):841–842, 2010.

[11] S. K. Sasse, M. Gruca, M. A. Allen, V. Kadiyala, T. Song, F. Gally, A. Gupta, M. A. Pufall, R. D. Dowell, and A. N. Gerber. Nascent transcript analysis of glucocorticoid crosstalk with TNF defines primary and cooperative inflammatory repression. *Genome Research*, 29:1753–1765, 2019.
