# Supplementary material for: Transcription factor enrichment analysis (TFEA) quantifies the activity of multiple transcription factors from a single experiment
Source: Commun Biol. 2021 Jun 2;4:661. doi: 10.1038/s42003-021-02153-7 (PMC8172830; doi:10.1038/s42003-021-02153-7)
Supplement: Supplementary file 2 — Description of Additional Supplementary Files [file 42003_2021_2153_MOESM2_ESM.pdf]

Description of Additional Supplementary Files

File name: Supplementary Data 1

Description: Accession numbers for data utilized to generate Figures in the paper (Figure 3, Figure 5, Figure 6, and Supplementary Figure 6), one tab per figure.
